# Unexpected diversity within the extinct elephant birds (Aves: Aepyornithidae) and a new identity for the world's largest bird

**DOI:** 10.1098/rsos.181295

**Published:** 2018-09-26

**Authors:** James P. Hansford, Samuel T. Turvey

**Affiliations:** 1 Institute of Zoology, Zoological Society of London, Regent's Park, London NW1 4RY, UK; 2 Ocean and Earth Science, National Oceanography Centre Southampton, University of Southampton, Waterfront Campus, European Way, Southampton SO14 3ZH, UK

**Keywords:** *Aepyornis*, Madagascar, megafauna, *Mullerornis*, multiple imputation, Quaternary extinction

## Abstract

Madagascar's now-extinct radiation of large-bodied ratites, the elephant birds (Aepyornithidae), has been subject to little modern research compared to the island's mammalian megafauna and other Late Quaternary giant birds. The family's convoluted and conflicting taxonomic history has hindered accurate interpretation of morphological diversity and has restricted modern research into their evolutionary history, biogeography and ecology. We present a new quantitative analysis of patterns of morphological diversity of aepyornithid skeletal elements, including material from all major global collections of aepyornithid skeletal remains, and constituting the first taxonomic reassessment of the family for over 50 years. Linear morphometric data collected from appendicular limb elements, and including nearly all type specimens, were examined using multivariate cluster analysis and the Bayesian information criterion, and with estimation of missing data using multiple imputation and expectation maximization algorithms. These analyses recover three distinct skeletal morphotypes within the Aepyornithidae. Two of these morphotypes are associated with the type specimens of the existing genera *Mullerornis* and *Aepyornis*, and represent small-bodied and medium-bodied aepyornithids, respectively. *Aepyornis* contains two distinct morphometric subgroups, which are identified as the largely allopatric species *A. hildebrandti* and *A. maximus.* The third morphotype, which has not previously been recognized as a distinct genus, is described as the novel taxon *Vorombe titan*. *Vorombe* represents the largest-bodied aepyornithid and is the world's largest bird, with a mean body mass of almost 650 kg. This new taxonomic framework for the Aepyornithidae provides an important new baseline for future studies of avian evolution and the Quaternary ecology of Madagascar.

## 1. Introduction

“When they found an Aepyornis with a thigh a yard long, they thought they had reached the top of the scale, and called him Aepyornis maximus. Then someone turned up another thigh-bone four feet six or more, and that they called Aepyornis titan … if they get any more Aepyornises, he reckons some scientific swell will go and burst a blood-vessel.”

H. G. Wells, *Aepyornis Island* [1]

An accurate understanding of taxonomy and diversity in recently extinct groups is necessary in order to understand evolutionary processes that have contributed to the functioning of past ecosystems, patterns of regional biogeography and ecological disruption caused by humans in prehistory [2,3]. However, current understanding of past diversity is often based on now-outdated and qualitative approaches, and as specimens on which original descriptions are based are often limited in number, they may not provide an accurate reflection of morphological diversity within and between extinct taxa [3,4]. Instability of nomenclature leads to taxonomic confusion and has serious implications for estimating past diversity and diversity change. Modern systematic approaches, using up-to-date quantitative methods, are necessary to review putative taxa and establish stable diversity estimates [5–7].

The Quaternary faunal record of Madagascar contains a unique and extraordinary megafauna, including giant lemurs, hippopotami, giant tortoises and the world's largest birds, the elephant birds. These taxa all survived into the Late Holocene and became extinct following the arrival of prehistoric human settlers, with available radiometric data suggesting that elephant birds persisted until around 1000 years ago [8]. Studies of the Malagasy megafauna have been dominated by the efforts of anthropologists investigating subfossil lemurs in tandem with studies of extant lemurs. Both giant tortoises and hippopotami have also been included in recent ecological reconstructions of Quaternary environments [9], but in comparison the radiation of elephant birds has seen remarkably little study since the advent of quantitative taxonomic methods involving multivariate analyses, so that the relationship between observed morphological diversity and the number of valid taxa within the group remains unclear.

## 2. History of research on elephant birds

Following the presentation and description of the world's largest egg and enormous avian skeletal remains from Madagascar in 1851 [10], the elephant birds (Aves: Aepyornithidae) [11] have excited debate in palaeontologists, archaeologists and zoologists ever since. These first specimens were reported to have a young geological age, which led to a series of nineteenth-century expeditions to find further subfossil remains of these giant birds and if possible extant individuals [12]. Although no living elephant birds were found, many additional skeletal and eggshell remains were discovered by subsequent researchers. Initial collections came from the extreme south and southwest of Madagascar, in swamp sites, coastal river sites and as part of alluvial deposition from rivers, and vast deposits of highly fragmented eggshells were found within coastal dune systems [10,13]. Towards the end of the nineteenth century, T. G. Rosaas collected further subfossil remains of elephant birds, hippopotami, giant tortoises and giant lemurs from Antsirabe, and passed these remains onto museum collections in Germany, Sweden, Norway, the UK and Austria [14].

Richard Owen investigated diversity within another extinct insular radiation of giant island-endemic ratites, the moa (Aves: Dinornithiformes) of New Zealand, through a series of linear measurements of leg bones (femora, tarsometatarsi and tibiotarsi) that allowed separation and diagnosis of moa taxa (e.g. total length; widths at proximal end, midshaft and distal end) [15,16]. These rudimentary linear morphometrics were subsequently used by other scientists studying elephant birds to establish an initial taxonomic framework for the Aepyornithidae during this early discovery period, but this was conducted through comparison of univariate measurements of incomparable elements (femur versus tarsometatarsus versus eggshell; [17]). These early attempts at taxonomic quantification, focused on allometric scaling, also had no realistic consideration of natural variation within taxa, and often interpreted marginally observable differences as being taxonomically important.

Throughout this initial discovery period, scientists in France, Britain and Germany erected 13 elephant bird species that were referred to three genera: *Aepyornis* Geoffroy Saint-Hilaire, 1851, type species *Aepyornis maximus* Geoffroy Saint-Hilaire, 1851 [10] (nine referred species); *Mullerornis* Milne-Edwards and Grandidier, 1894 [18], type species *Mullerornis betsilei* [19] (three referred species); and *Flacourtia* Andrews, 1895, type species *Mullerornis rudis* [20] (one referred species) (table 1). Published descriptions of these taxa were based almost entirely on the most common elements found, the robust leg bones, as well as upon major size differences between the two most widely accepted genera, *Aepyornis* (approx. 400 kg) and *Mullerornis* (approx. 100 kg). Differentiation of species was based largely on linear measurements of the limited remains then available for study in respective national collections and via inter-museum loans of casts. Most of these taxa were erected between 1893 and 1895, and authors attempted to demonstrate their authority by synonymizing ‘competing’ taxa, often focusing on laying claim to the largest birds (with *A. maximus* Geoffroy Saint-Hilaire, 1851, *A. ingens* Milne-Edwards and Grandidier, 1894 [18] and *A. titan* Andrews, 1894 [22] all variously reported as being the largest in size). This ‘conflict of authority’ [26] led to extreme confusion over diversity within the family, and also over biogeographical patterns shown by aepyornithids across the vast and highly variable ecological regions of Madagascar. While most (although not all) of the referred type series associated with proposed taxa can be identified for study today, few holotypes were identified in original publications, and several species are known from syntype series comprising multiple elements that were not necessarily from the same taxon (table 2), further adding to taxonomic confusion.

**Table 1.** Nomenclaturally valid species of elephant birds. Note: *M. rudis* was subsequently designated as the type species of *Flacourtia* by Andrews [20].

putative species	author	revised species (after Brodkorb)	distribution (after Brodkorb)
*A. maximus*	Geoffroy Saint-Hilaire, 1851 [10]	*A. maximus*	Ambolisatra, Masikoro, between Belo-sur-Mer and Morondava, Itampulu Vé, Lamboharana
*A. modestus*	Milne-Edwards and Grandidier, 1869 [21]	*A. maximus*	
*A. titan*	Andrews, 1894 [22]	*A. maximus*	
*A. ingens*	Milne-Edwards and Grandidier, 1894 [18]	*A. maximus*	
*A. grandidieri*	Rowley, 1867 [17]	*A. medius*	Cape Sainte-Marie, between Belo-sur-Mer and Morondava
*A. medius*	Milne-Edwards and Grandidier, 1869 [21]	*A. medius*	
*A. cursor*	Milne-Edwards and Grandidier, 1894 [18]	*A. medius*	
*A. lentus*	Milne-Edwards and Grandidier, 1894 [18]	*A. medius*	
*A. hildebrandti*	Burckhardt, 1893 [23]	*A. hildebrandti*	Antsirabé
*A. mulleri*	Milne-Edwards and Grandidier, 1894 [18]	*A. hildebrandti*	
*A. gracilis*	Monnier, 1913 [24]	*A. gracilis*	unknown
*M. betsilei*	Milne-Edwards and Grandidier, 1894 [18]	*M. betsilei*	Antsirabé
*M. agilis*	Milne-Edwards and Grandidier, 1894 [18]	*M. agilis*	near Morondava
*M. rudis*	Milne-Edwards and Grandidier, 1894 [18]	*M. rudis*	between Belo-sur-Mer and Morondava
*M. grandis*	Lamberton, 1934 [25]	n.a.	n.a.
total: two genera, 15 species		total: two genera, seven species

**Table 2.** Taxonomic matrix describing morphometric assignment and taxonomic seniority of type material of named elephant bird species to the morphometric clusters identified in this study.

cluster	type specimens included within each cluster			other type specimens referable to cluster based on published measurements	senior synonym for cluster	available senior genus name for cluster	name assigned to taxon
	femur	tibiotarsus	tarsometatarsus				
1	(1) *Aepyornis modestus* 1869, (2) *Aepyornis hildebrandti* 1893 (part of type series)	(1) *Mullerornis agilis* 1894, (2) *Mullerornis rudis* 1894	—	(1) *Mullerornis betsilei* 1894	*Aepyornis modestus* 1869	*Mullerornis* 1894	*Mullerornis modestus* (Milne-Edwards and Grandidier, 1869)
2a	(1) *Aepyornis hildebrandti* 1893 (part of type series), (2) *Aepyornis gracilis* 1912	—	(1) *Aepyornis hildebrandti* 1893 (part of type series), (2) *Aepyornis lentus* 1894		*Aepyornis hildebrandti* 1893 (based on well-predicted tarsometatarsus, not poorly predicted femur)	*Aepyornis* 1851 (name available for entirety of cluster 2, with reference to assessment of data for cluster 2b)	*Aepyornis hildebrandti* Burckhardt, 1893
2b	(1) *Aepyornis medius* 1869	—	(1) *Aepyornis cursor* 1894	(1) *Aepyornis maximus* 1851	*Aepyornis maximus* 1851	*Aepyornis* 1851	*Aepyornis maximus* Geoffroy Saint-Hilaire, 1851
3	(1) *Aepyornis titan* 1894 (part of type series)	(1) *Aepyornis titan* 1894 (part of type series), (2) *Aepyornis ingens* 1894 (part of type series)	(1) *Aepyornis ingens* 1894 (part of type series)		*Aepyornis titan* 1894	None available; new genus name *Vorombe* erected to describe this cluster (see text)	*Vorombe titan* (Andrews, 1894)

In the early twentieth century, further attempts to clarify the taxonomic diversity of the Aepyornithidae were made by Monnier [24], Lambrecht [27] and Lamberton [25]. These later researchers had access to large collections in France and Madagascar to help describe taxa more accurately, including cranial series and articulated skeletons, but they still failed to consider variation within species adequately, as their definitions were limited by the small series of adult specimens of femora, tibiotarsi and tarsometatarsi available for study for many taxa. Whilst Monnier and Lamberton both erected new putative elephant bird species during their reviews, bringing the total number of named species to 15 by 1934, the results of these efforts saw several taxa originally described from incomparable elements and based on approximate size comparisons to now become reduced to the status of junior synonyms. This framework of reduced elephant bird diversity (two genera, seven species: *Aepyornis*, four species; *Mullerornis*, three species) was summarized by Brodkorb [28]. Although his review did not include all previously described elephant bird taxa (*M. grandis* Lamberton, 1934 [25], based on material then curated in Madagascar, was not considered), it is still the most commonly cited framework for species-level nomenclature of aepyornithids in modern literature, biogeographical studies and phylogenetic analysis [14,29–31] (table 1). Brodkorb's qualitative assessment of species distributions within *Aepyornis* recognized geographical co-occurrence of *A. maximus* and *A. medius* in both the central west coast region and the extreme south of Madagascar, with *A. hildebrandti* Burckhardt, 1893 [23] found in the central highlands. *Mullerornis* was also considered to contain two geographically co-occurring species, *M. agilis* Milne-Edwards and Grandidier, 1894 [18] and *M. rudis* Milne-Edwards and Grandidier, 1894 [18], with the area of their spatial overlap limited to the central west coast region near Belo-sur-mer and Morondava, and with a third recognized species, *M. betsilei* Milne-Edwards and Grandidier, 1894 [18], restricted to the central highlands.

The elephant birds have been the focus of remarkably little study during the late twentieth and early twenty-first centuries in comparison to moa and many other Quaternary megafaunal vertebrates. Following the recent development of methods of evolutionary and ecological analysis using ancient biomolecules, elephant bird material has been studied in efforts to reconstruct their evolutionary history and phylogenetic relationships [32], dietary ecology [33] and causes of extinction [34]. In particular, aepyornithid ancient DNA sequence data have been used to infer the timing of divergences between sampled taxa, estimated to be 27.6 Ma between material assigned to *Mullerornis agilis* and *Aepyornis hildebrandti*, and 3.3 Ma between *A. hildebrandti* and *A. maximus* [29,30]*.* However, this research has been conducted using either skeletal samples of uncertain taxonomic identification [29], combined sequences from specimens with varying morphology [31] or eggshell fragments from coastal dune sites and archaeological assemblages which are typically not associated with adult or juvenile skeletal remains [30,32]. Aepyornithid eggshell fragments exhibit differences in thickness that are interpreted as representing two distinct size categories, which have been associated with the two currently recognized genera *Aepyornis* (approx. 4 mm thick) and *Mullerornis* (approx. 2 mm thick) [30]. These phylogenetic assumptions are therefore difficult to interpret in the context of aepyornithid taxonomy, which is based almost entirely upon morphology of skeletal elements rather than eggshell.

## 3. Towards a modern morphometric framework for elephant bird taxonomy

Multivariate analysis of morphometric data derived from skeletal elements constitutes a significantly more powerful diagnostic tool for delimiting taxa than the univariate and bivariate methods used in historical aepyornithid systematic studies. However, multivariate methods require data frames with no missing values. As aepyornithid remains are rarely found completely intact, attempts to quantify multivariate morphometric data inclusive of all available specimens must compensate for these data gaps [35–37].

Omission of characters and specimens from analysis is a common method for addressing the problem of missing data [4,37,38]. However, this approach can lead to underrepresentation of the morphological diversity present in specimen assemblages and can also affect statistical robustness of analyses. Maximization of datasets through a stepped process of incrementally omitting specimens or characters with the largest number of missing data points can also produce alternate datasets with the same quantity of data, but may omit specimens that represent cryptic taxa, or key diagnostic features [37,39].

The alternative to omitting data is to estimate missing values while preserving natural variation of characters within taxa. One approach, imputation based on the means of observed variables, can create conservative models that can underrepresent natural variation within the morphological range for a given taxon [39] and may generate composite means from data that combine separate morphologically distinct taxa. In comparison, multiple imputation (MI) methods are robust to these potential sources of error, and even against anatomically and taxonomically biased data gaps in morphometric analyses [40]. Comparative analysis of available methods indicates that MI using expectation maximization (EM) algorithms constitutes an effective compromise between accuracy of imputation and coverage probability [39].

Many studies that aim to test the validity of a given taxonomic hypothesis using morphometric data are supported by a well-delimited higher-order nomenclature and good geographical provenance of specimens [32,41,42]. Conversely, the poorly defined taxonomy of the Aepyornithidae necessitates an unsupervised, objective exploration of morphotype clusters within the multidimensional shape-space generated from multiple linear measurements, to identify the most parsimonious solution for clustering morphotypes in order to determine specimen group assignment.

To clarify the confused state of elephant bird taxonomy, and to assess how many taxonomic units represented by distinct morphological clusters can be identified within a rigorously determined quantitative framework, we performed a series of morphometric analyses on linear measurement data from almost all of the specimens of aepyornithid appendicular limb elements available for study in global museum collections. We used an iterative modelling approach to permit comparison between models alternately assigning specimens to a varying number of clusters [43]. This study constitutes the first detailed revision of elephant bird taxonomy for over half a century and the first rigorous quantitative study of intraspecific variation and diagnostic morphological characters within aepyornithids, and permits formal reassessment of taxonomic diversity within this evolutionarily important but under-studied extinct avian family.

## 4. Material and methods

### 4.1. Specimens and measurements

**Figure 1.**
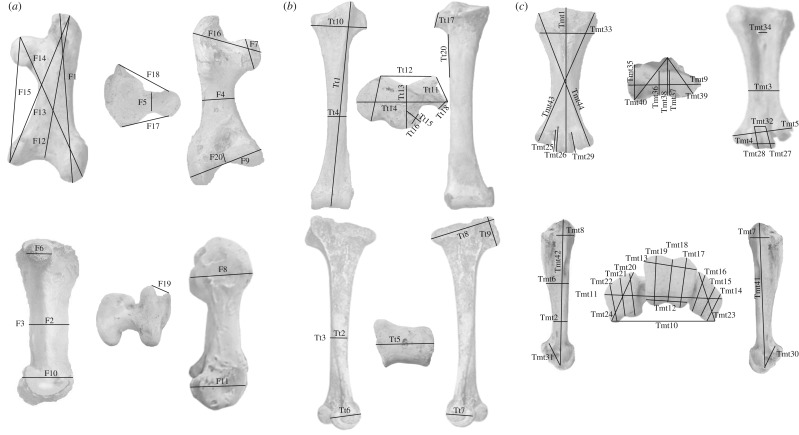
Diagram of linear measurements taken on aepyornithid leg bones. (*a*) Femoral measurements. F1: total length; F2: minimum midshaft width; F3: circumference at minimum midshaft width; F4: maximum midshaft width; F5: circumference of caput femoris; F6: dorsoventral diameter of caput femoris; F7: proximo-distal diameter of caput femoris; F8: dorsoventral thickness of trochanter femoris; F9: maximum width of distal condyles; F10: maximum height of condylus medialis; F11: maximum height of condylus lateralis; F12: sulcus patellaris to trochanter femoris; F13: trochanter femoris to condylus medialis; F14: proximo-medial extreme of caput femoris to condylus lateralis; F16: medio-lateral length of caput femoris; F17: dorsal extremity of crista trochanteris to dorsal extremity of caput femoris; F18: ventral extremity of crista trochanteris to ventral extremity of caput femoris; F19: trochlea fibularis width; F20: distance between medial and condylus lateralis. (*b*) Tibiotarsal measurements. Tt1: total length; Tt2: minimum midshaft width; Tt3: circumference at Tt2; Tt4: maximum midshaft width; Tt5: width of condyles; Tt6: maximum height, condylus medialis; Tt7: maximum height, condylus lateralis; Tt8: maximum width of head, including crest; Tt9: width of proximal end, including crista cnemialis cranialis; Tt10: width of head; Tt11: distance between cnemial crests; Tt12: extreme width of posterior groove; Tt13: posterior groove to external condyle; Tt14: posterior groove height to external condyle; Tt15: external condyle width; Tt16: external condyle height; Tt17: outer cnemial crest width; Tt18: outer crista cnemialis lateralis height; Tt19: total outer crista cnemialis lateralis ridge length; Tt20: tibia scar. (*c*) Tarsometatarsal measurements. Tmt1: length; Tmt2: minimum shaft thickness (not midshaft); Tmt3: shaft width at Tmt2; Tmt4: trochlea III width; Tmt5: width (all trochleae); Tmt6: head height at midpoint, including ridge; Tmt7: maximum height, proximal end of metatarsal II; Tmt8: maximum height, proximal end of metatarsal IV; Tmt9: head width; Tmt10: inside curve (plantar) across three trochleae; Tmt11: outside curve (cranial) across three trochleae; Tmt12: trochlea III, plantar width; Tmt13: trochlea III, cranial width; Tmt14: trochlea II, medial thickness; Tmt15: trochlea II, central thickness; Tmt16: trochlea II, lateral thickness; Tmt17: trochleae III, medial thickness; Tmt18: trochlea III, central thickness; Tmt19: trochleae III, lateral thickness; Tmt20: trochlea IV, medial thickness; Tmt21: trochlea IV, central thickness; Tmt22: trochlea IV, lateral thickness; Tmt23: trochlea III, diagonal length; Tmt24: trochlea IV, diagonal length; Tmt25: trochlea III, medial length, outside to notch; Tmt26: trochlea III, medial length, outside with notch; Tmt27: trochlea III, medio-cranial length; Tmt28: trochlea III, lateral-cranial length; Tmt29: trochlea III, lateral length, outside; Tmt30: trochlea II length; Tmt31: trochlea IV length; Tmt32: trochlea III, cranial (peak to peak) notch width; Tmt33: total width at foramina; Tmt34: foramina width; Tmt35: maximum anterior–posterior depth of external cotyle; Tmt36: minimum depth of head; Tmt37: maximum depth at hypotarsal ridge (no ridge); Tmt38: maximum depth at hypotarsal ridge (inclusive of ridge); Tmt39: proximal–lateral extreme of head to hypotarsal ridge extreme; Tmt40: proximal–medial extreme of head to hypotarsal ridge extreme; Tmt41: length, trochlea II to head; Tmt42: length, trochlea IV to head; Tmt43: diagonal length, trochlea II to head; Tmt44: diagonal length, trochlea IV to head.

Aepyornithid femora (*n* = 97), tibiotarsi (*n* = 162) and tarsometatarsi (*n* = 87) of adult individuals (defined on the basis of full fusion of epiphyses) were studied from the following collections: American Museum of Natural History, USA (AMNH), Centre ValBio, Madagascar (CVB), Museum für Naturkunde, Germany (MfN), Museum National d'Histoire Naturelle, France (MNHN), Natural History Museum, UK (NHMUK), Naturhistorisches Museum, Austria (NHMW), Oxford University Museum, UK (OUMNH), Université d'Antananarivo, Madagascar (UA), Natural History Museum, University of Oslo, Norway (UIO), United States National Museum, USA (USNM) and Zoologiska Museum, Uppsala Universitet, Sweden (ZIUU) (electronic supplementary material, table S1). A standard series of 20 femoral, 20 tibiotarsal and 44 tarsometatarsal measurements were taken where possible (figure 1). Measurements up to 150 mm were taken using dial callipers accurate to 0.02 mm. Circumference and measurements of more than 150 mm were made using a measuring tape accurate to 1 mm.

Five described species could not be included directly in this analysis. The type material of *Mullerornis grandis* Lamberton, 1934 was lost in a fire in 1995, and the skeletal type material of *Aepyornis maximus* Geoffroy Saint-Hilaire, 1851, *Mullerornis betsilei* Milne-Edwards and Grandidier, 1894 and *Aepyornis mulleri* Milne-Edwards and Grandidier, 1894 cannot now be located in museum collections, meaning that type specimens for these species could not be included in the long bone measurement dataset. *Aepyornis grandidieri* Rowley, 1867 was described from eggshell remains only and therefore cannot be compared to other taxa.

### 4.2. Missing data imputation

Of the total dataset of 346 specimens, only 82 specimens (19 femora, 42 tibiotarsi and 21 tarsometatarsi) were completely intact and undamaged (electronic supplementary material, table S1). As some taxa might only be represented by broken specimens, proportions of missing linear measurements from broken specimens were examined in 5% stepped increments. Selection of first-round data frames was defined by the inclusion of elements with less than 25% of linear measurements missing (approx. 50% of available specimens) to minimize imputation and maximize potential taxonomic inclusion. Skeletal elements with more than 25% of linear measurements missing were omitted from the first round of imputation calculations and taxonomic assessments. The first-round data frames included 48 femora (49% of specimens and 11.6% imputed data), 73 tibiotarsi (45% of specimens and 7.8% imputed data) and 46 tarsometatarsi (53% of specimens and 5.8% imputed data; electronic supplementary material, table S2).

All statistical analysis was performed in R v. 3.1.3 [44]. MI methods using EM algorithms were used to estimate the linear measurements of missing portions of elements to create a complete data frame using the ‘ImputePCA’ function of the MissMDA package. The first round of the algorithm imputed missing data using the mean of the variable across the observed values, and a principal component analysis (PCA) was performed on this imputed data frame. Values fitted by the PCA were then used to predict new values for missing data, while retaining observed values. The process of parameter estimation via PCA and refitting of imputed values were then repeated until the predicted missing values were converged. This method provides good estimations of the missing data as there were very strong correlations between observed variables, and in the first round, the number of missing values was small. However, to remove the problem of overfitting through EM algorithms, we used *k*-fold cross-validation as a regulation mechanism to remove noise and improve prediction quality. The ‘tuned parameters’ were determined by fivefold cross-validation to find the PCA loadings that produced the smallest mean square error of predictions, using the ‘estim_ncpPCA’ function of the MissMDA package [45]. Linear measurement data were scaled to their unit variance by subtracting the feature mean from the individual feature value and then dividing by the feature's standard deviation, to mitigate the overemphasis of variation in overall size on PCA analyses.

### 4.3. Cluster analysis

PCA was conducted on observed and imputed data derived from the first round of EM imputation, to investigate whether morphometric measurement data are able to identify discrete clusters of elephant bird specimens that are likely to correspond with taxonomically distinct groups. This approach extracts and summarizes the major features of morphometric shape variation and reduces high dimensionality to examine the distribution of different taxonomic groups in shape-space, without making any prior assumptions about the pattern of clustering of specimens.

The package ‘MClust’ [46] was used to perform hierarchical model-based classification cluster analysis, based on PCA loadings derived from the first-round observed and imputed datasets. Selection of the most likely model was based on Schwarz's Bayesian information criterion (BIC) [47]. BIC is determined by the value of the maximized log-likelihood model, penalized by an increasing number of model parameters and allowing the comparison of models with differing numbers of clusters and representation in morphospace (equal and unequal variance; spherical, diagonal and ellipsoidal shape; and equal and varying volume). PCA loadings included in the cluster model were introduced in a stepped sequence until BIC was able to identify a distinct pattern. Specimens demonstrating high levels of classification uncertainty (0.05 and above) were removed from the first-round dataset and added to the dataset with more than 25% missing data. As BIC weights against an increasing number of groups, we first obtained the highest number of clusters from each element (unsupervised clustering) and then if necessary re-clustered the data based on fixed numbers of clusters obtained for other limb elements (supervised clustering) to determine whether a stable result could be observed.

A second dataset was generated to include all data with more than 25% missing specimens, any available type specimens that had not yet been included in cluster models, and all specimens with location data. Missing data point imputation, clustering, and removal of specimens demonstrating high levels of classification uncertainty were then performed. Clustering was performed using the same method as above, but the number of clusters was limited to the number observed from the first round of analysis.

The large amount of missing data included in this second phase of imputation means that cluster assignment of these poorer-quality specimens must be interpreted with caution; however, this represents the only quantitative framework for identifying distinct morphological forms from incomplete remains of elephant birds. Where accession data were available for specimens, their cluster and geographical location was recorded to examine any potential pattern of spatial distributions. This second dataset included 64 femora (26.9% missing datapoints), 95 tibiotarsi (22.8% missing datapoints) and 70 tarsometatarsi (27.0% missing datapoints) (electronic supplementary material, table S2).

All PCA analyses were re-run using log-transformed data, to further reduce the potential confounding influence of variation in size alone [48].

### 4.4. Summary statistics

ANOVAs were performed on individual measurements for each morphological cluster of femora, tibiotarsi and tarsometatarsi, in order to describe the measurement parameters of each cluster and therefore define the taxonomic groupings represented by each cluster. Mass estimations were calculated using the Campbell and Marcus algorithm for estimating body mass in birds from femoral least-shaft circumference (LogM = 2.411 × LogLCF − 0.065) [49]. Mean mass and standard deviation were determined for each cluster, based on observed data only.

### 4.5. Radiometric dating

Bone samples from elephant bird specimens assigned to different morphometric clusters were submitted for accelerator mass spectrometer (AMS) ^14^C dating at the Oxford Radiocarbon Accelerator Unit, Oxford, UK and calibrated using ShCal13 [50] implemented in OxCal 4.1 [51].

## 5. Results

### 5.1. Morphometric analysis

From our sample, 41 femora, 83 tibiotarsi and 41 tarsometatarsi were excluded from the first round of analysis due to exceeding the more than 25% missing marker criterion for taxonomic assessment. The percentage of total imputed data generated in this round was 11.6% for femora, 9.1% for tibiotarsi and 5.5% for tarsometatarsi. Five femora, 49 tibiotarsi and one tarsometatarsus were excluded from taxonomic classification through clustering due to high uncertainty in classification (greater than 5% uncertainty). Four femora, 43 tibiotarsi and 12 tarsometatarsi were excluded from subsequent biogeographical analysis, again due to greater than 5% uncertainty of classification. For the biogeographical assessment dataset, 26.9% of femoral markers, 23.0% of tibiotarsal markers and 27.0% of tarsometatarsal markers were not observed and so were imputed.

**Figure 2.**
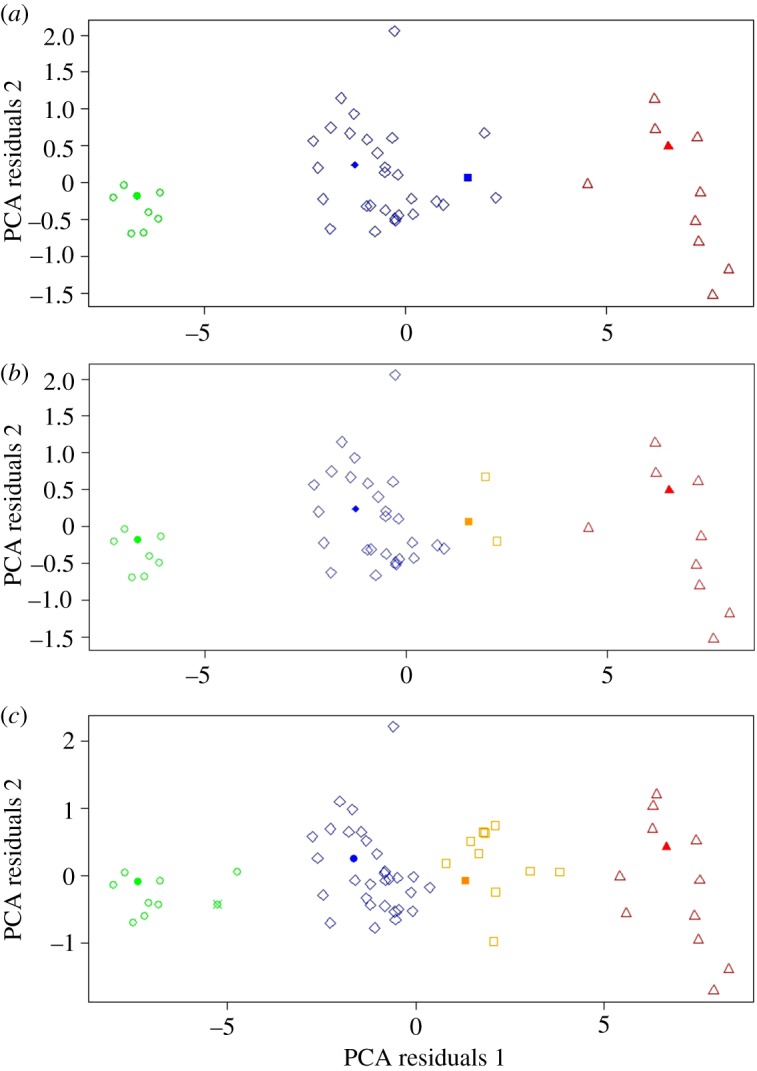
(*a*) Unsupervised clusters of femora, less than 25% missing data. PCA axis 1, 90.8%; PCA axis 2, 2.1%. Cluster 1 = circles, cluster 2a = diamonds, cluster 2b = squares, cluster 3 = triangles. Type specimens/series: filled circle, *A. modestus*; filled diamond, *A. gracilis*; filled square, *A. medius*; filled triangle, *A. titan.* (*b*) Supervised clusters of femora (four possible groups only), less than 25% missing data. PCA axis 1, 90.8%; PCA axis 2, 2.1%. Cluster 1 = circles, cluster 2a = diamonds, cluster 2b = squares, cluster 3 = triangles. Type specimens/series: filled circle, *A. modestus*; filled diamond, *A. gracilis*; filled square, *A. medius*; filled triangle, *A. titan.* (*c*) Unsupervised clusters of femora, more than 25% missing data. PCA axis 1, 91.4%; PCA axis 2, 2.1%. Cluster 1 = circles, cluster 2a = diamonds, cluster 2b = squares, cluster 3 = triangles. Type specimens/series: filled circle, *A. modestus*; quartered circle, *A. hildebrandti*; filled diamond, *A. gracilis*; filled square, *A. medius*; filled triangle, *A. titan.*

**Figure 3.**
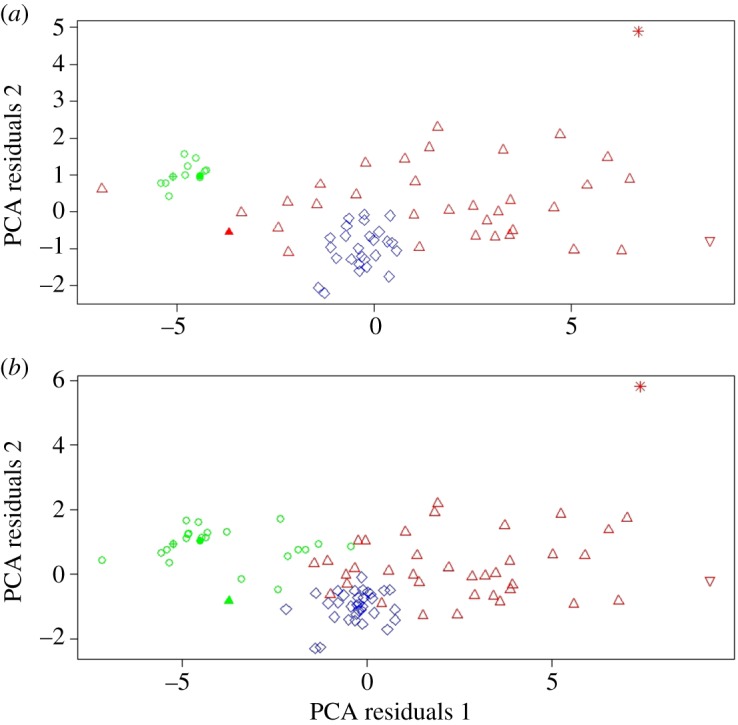
(*a*) Unsupervised clusters of tibiotarsi, less than 25% missing data. PCA axis 1, 53.7%; PCA axis 2, 7.4%. Cluster 1 = circles, cluster 2 = diamonds, cluster 3 = triangles. Type specimens/series: filled circle, *M. agilis*; crossed circle, *M. rudis*; filled triangle, *A. hildebrandti*; upside-down triangle, *A. ingens*; star, *A. titan*. (*b*) Unsupervised clusters of tibiotarsi, more than 25% missing data. PCA axis 1, 67.2%; PCA axis 2, 6.6%. Cluster 1 = circles, cluster 2 = diamonds, cluster 3 = triangles. Type specimens/series: filled circle, *M. agilis*; crossed circle, *M. rudis*; filled triangle, *A. hildebrandti*; upside-down triangle, *A. ingens*; star, *A. titan*.

**Figure 4.**
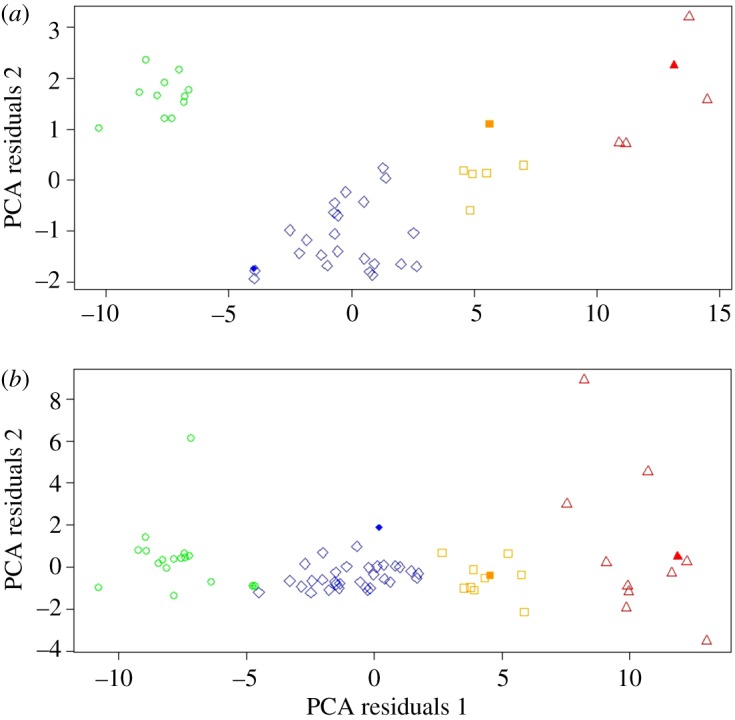
(*a*) Unsupervised clusters of tarsometatarsi, less than 25% missing data. PCA axis 1, 86.8%; PCA axis 2, 4.6%. Cluster 1 = circles, cluster 2a = diamonds, cluster 2b = squares, cluster 3 = triangles. Type specimens/series: filled diamond, *A. hildebrandti*; filled square, *A. cursor*; filled triangle, *A. ingens*. (*b*) Unsupervised clusters of tarsometatarsi, more than 25% missing data. PCA axis 1, 82.7%; PCA axis 2, 6.6%. Cluster 1 = circles, cluster 2a = diamonds, cluster 2b = squares, cluster 3 = triangles. Type specimens/series: crossed circle, *A. hildebrandti*; filled diamond, *A. lentus*; filled square, *A. cursor*; filled triangle, *A. ingens*.

Cluster analysis performed separately on PCA weightings created from each specimen's linear measurements from all three limb bones revealed that the comprehensive sample of elephant bird specimens analysed in this study fall into multiple distinct morphometric groups, defined as a stable result by BIC differentiation between cluster models of greater than 2 (electronic supplementary material, table S2). Femora (figure 2) and tibiotarsi (figure 3) both demonstrated stable clustering into three distinct groups. Femora required two principal components to achieve a stable cluster model, whereas tibiotarsi required only one principal component. The tarsometatarsal dataset required four principal components to achieve a stable result and clustered into four distinct groups (figure 4). As BIC weights against increasing numbers of groups, supervised clustering based on four possible groups (as determined by tarsometatarsal clustering) was then applied to both the femoral and tibiotarsal data, to investigate whether further subclustering could also be identified within the three primary clusters for these elements. The femoral dataset subdivided cluster 2 into two further subgroups (figure 2), but the tibiotarsal dataset was unable to identify any further subdivision within its sample. The tibiotarsal dataset had poorly defined clusters and the weakest predictive power for defining morphotypes.

In all taxonomic PCA clusters, PC1 was highly correlated (greater than 0.75) with almost all measurements from each skeletal element (although not with TT5 or TT20), indicating a primary separation of clusters based on overall size (electronic supplementary material, table S2). However, clusters overlap in size ranges and can also be differentiated by other patterns of distinctive morphotype variation, with clear autocorrelation between size and differing morphology.

In cluster analysis of log-transformed data, femoral data were more stable than tarsometatarsal data. Four stable groups were recovered in unsupervised clustering of femoral data: clusters 1, 2 and 3 were separated along PC1, and cluster 2 was further subdivided into two subclusters on PC2. When supervised clustering based on these four groups was then applied to tarsometatarsal data, this produced the same classification of all tarsometatarsal specimens within the same clusters as in non-transformed cluster analysis (electronic supplementary material, figure S1 and table S3).

### 5.2. Taxonomy of morphometric clusters

Tarsometatarsal data provide the best-resolved assessment of morphological diversity within aepyornithids, as the four clusters based on data for this limb bone resolve well and group membership is the most stable (figure 4). Femoral data predict three groups as the most parsimonious result of clustering analysis, but also demonstrate well-resolved clusters and stable group membership when restricted to four possible clusters (figure 2). Tibiotarsal data also only predict three clusters, although morphological diversity on the basis of tibiotarsal data is not represented well by our current measurement framework, and the only consistent differences that can be established between samples are upon extremely large size differences when large amounts of data are compared (figure 3).

We interpret the three clusters identified using femoral and tarsometatarsal data as representing genus-level differentiation, and the two consistent and stable subgroups within cluster 2 shown by femora and tarsometatarsi as representing further species-level differentiation within this cluster. Three existing generic names are available in the published literature that may correspond to some or all of the clusters identified in this analysis: *Aepyornis* Geoffroy Saint-Hilaire, 1851; *Mullerornis* Milne-Edwards and Grandidier, 1894 and *Flacourtia* Andrews, 1895. The type specimens or type series of 10 of the 15 species that have been assigned to these genera can still be located in museum collections, and were included within the clustering analysis. The taxonomic identity of each morphometric cluster was established by determining which type specimens were included within which clusters, and which of these type specimens represented the oldest available taxonomic name (table 2). Specimens that demonstrated high probability of conflicting cluster classification (high uncertainty) were excluded from taxonomic conclusions.

Cluster 1 represents the smallest specimens across all skeletal element datasets and contains the type material of *Aepyornis modestus* Milne-Edwards and Grandidier, 1869 (holotype: femur), *Mullerornis agilis* Milne-Edwards and Grandidier, 1894 (part of syntype series: tibiotarsus) and *Mullerornis rudis* Milne-Edwards and Grandidier, 1894 (part of syntype series: tibiotarsus). Cluster 2 contains the intermediate-sized specimens across all skeletal element datasets and contains well-predicted type material of *Aepyornis hildebrandti* Burckhardt, 1893 (part of syntype series: tarsometatarsus), *Aepyornis medius* Milne-Edwards and Grandidier, 1869 (holotype: femur), *Aepyornis cursor* Milne-Edwards and Grandidier, 1894 (holotype: tarsometatarsus), *Aepyornis lentus* Milne-Edwards and Grandidier, 1894 (holotype: tarsometatarsus) and *Aepyornis gracilis* Monnier, 1913 (holotype: femur). When subdivided by supervised cluster classification (four groups), cluster 2a contains the type material of *Aepyornis hildebrandti* (part of syntype series: tarsometatarsus), *Aepyornis lentus* and *Aepyornis gracilis*. The syntype femur of *Aepyornis hildebrandti* was also assigned to cluster 2a, but this specimen is incomplete and cluster assignment was poorly predicted due to high uncertainty, so taxonomic assignment of the name *Aepyornis hildebrandti* was based solely on the tarsometatarsus. Cluster 2b contained the holotypes of *Aepyornis medius* and *Aepyornis cursor.* All tibiotarsi that fell within cluster 2 demonstrated high uncertainty of cluster classification and were therefore not used for taxonomic assessment. Cluster 3 contains the largest specimens of all skeletal elements and contains the syntype material of *Aepyornis titan* Andrews, 1894 (femur, tibiotarsus) and *Aepyornis ingens* Milne-Edwards and Grandidier, 1894 (tibiotarsus and tarsometatarsus).

Owing to the vague description of skeletal measurements, only eggshell dimensions can be compared accurately from the original description of *Aepyornis maximus* by Geoffroy Saint-Hilaire [10]. However, Owen [52] published a small number of measurements of the incomplete tarsometatarsus originally reported as part of the type series for this species by Geoffroy Saint-Hilaire. Other measurement ranges for this taxon are available in Monnier [24], but are based on a range of specimens that Monnier regarded as constituting the same species, rather than from the syntype tarsometatarsus. The only measurement provided by Owen [52] that can be compared to our dataset is the extreme breadth across the trochlear condyles. This is a discrete (non-overlapping) measurement for the clusters presented here (cluster 1: 65–79.24 mm, cluster 2a: 105–118 mm, cluster 2b: 125.18–140.2 mm, cluster 3: 164–178 mm). The measurement value for *A. maximus* as reported by Owen is 127 mm, indicating that the type series of this species falls within the range of cluster 2b.

The original published description of the *Mullerornis betsilei* type series by Milne-Edwards & Grandidier [18] includes four measurements from the tarsometatarsus (length: 310 mm, circumference: 80 mm, width of shaft: 27 mm, proximal width: 70 mm) and five from the tibiotarsus (length: 390 mm, shaft circumference: 90 mm, width: 30 mm, proximal width: 75 mm, distal width: 60 mm). Tarsometatarsal length cannot be used alone to diagnose taxa, as there is considerable overlap in this measurement between clusters. Using the proximal width of the tarsometatarsus, which exhibits discrete measurement values between clusters (cluster 1: 65.8–81.46 mm, cluster 2a: 99.7–123.1 mm, cluster 2b: 140.3–150.5 mm, cluster 3: 173–184 mm), the value reported for *M. betsilei* (70 mm) falls within the range of cluster 1.

Lamberton [25] included ranges for six femoral and seven tibiotarsal measurements in the description of *Mullerornis grandis*. Here, we use the minimum femoral shaft circumference, which shows discrete measurement values between clusters for *Mullerornis* (cluster 1: 114–158 mm, cluster 2a: 172–210 mm, cluster 2b: 208–254 mm, cluster 3: 253–288 mm). The minimum shaft circumference range reported for *M. grandis* (125–145 mm) falls within the upper range of cluster 1.

*Aepyornis mulleri* was described on the basis of a skull, mandible, vertebrae, ribs, sternum, part of pelvis, ‘the leg bones’ and phalanges [18]. No published measurement data exist for the ‘leg bones’, and so this species cannot be assigned to any of our postcranial morphometric clusters based on comparative measurements. *A. mulleri* was previously considered to be a subjective synonym of *A. hildebrandti* by Monnier [24], but is not considered further in this quantitative taxonomic assessment.

The genus *Aepyornis* Geoffroy Saint-Hilaire, 1851 was first used to describe *Aepyornis maximus*, which our data demonstrate can be assigned to cluster 2b, and this name can therefore be interpreted as the senior synonym for all of cluster 2. The genus *Mullerornis* Milne-Edwards and Grandidier, 1894 was first used to describe *Mullerornis betsilei*, which was subsequently designated as the type species by Richmond [19], and which is assigned to cluster 1. As two of our clusters correspond to different genera previously defined by earlier authors on the basis of qualitative or univariate assessment of variation within the Aepyornithidae [10,18], this supports our interpretation of all three primary clusters in our analysis as representing genus-level differentiation. Cluster 3, which contains specimens that were originally assigned to two species of *Aepyornis* in 1894, represents a further distinct morphotype which on this basis also needs to be recognized as distinct at the genus level. A third aepyornithid genus name, *Flacourtia* Andrews, 1895, is also available, but the holotype tibiotarsus of the type species *Mullerornis rudis* clusters reliably within cluster 1, and so the name *Flacourtia* represents a junior synonym of *Mullerornis* and cannot be used to describe cluster 3. There is therefore no available genus name that can be applied to cluster 3.

Our analysis does not distinguish distinct morphotypes within cluster 1 (*Mullerornis*), and so we apply the oldest species name for this cluster, *Aepyornis modestus*, to name the single species that can be recognized in this genus. Cluster 2 (*Aepyornis*) can be separated into two distinct morphological groups on the basis of both tarsometatarsal and femoral data, and we interpret these groups as representing separate species within the same genus: the oldest available species names within each cluster are *Aepyornis hildebrandti* (cluster 2a) and *Aepyornis maximus* (cluster 2b). No morphological differentiation can be demonstrated within cluster 3 (unnamed genus). Within this cluster, the two species names *Aepyornis titan* and *Aepyornis ingens* were both published in 1894, but *titan* (published January 1894) predates *ingens* (published February 1894) by one month, so that the oldest available species name for this group is *Aepyornis titan.* Body mass estimates for these four recognized aepyornithid taxa are given in table 3, and measurement datasets are given in tables 4–6.

**Table 3.** Mass estimates for elephant bird species recognized in this study.

femoral mass estimation (kg)	*Mullerornis modestus*	*Aepyornis hildebrandti*	*Aepyornis maximus*	*Vorombe titan*
maximum	172	342	541	732
minimum	78	211	334	536
mean	107.7	283.15	409.5	642.9
standard deviation	33.2	34.1	71.8	62.6
sample size	10	29	8	10

**Table 4.** Femoral measurement ranges for elephant bird species recognized in this study.

measurement	*Mullerornis modestus*		*Aepyornis hildebrandti*		*Aepyornis maximus*		*Vorombe titan*	
	range (mm)	*N*	range (mm)	*N*	range (mm)	*N*	range (mm)	*N*
F1	245–268	5	307.0–347.0	18	354.0–383.0	4	437.0–490.0	8
F2	28.4–43.2	10	45.9–57.7	29	51.3–69.5	8	64.4–94.1	12
F3	114–158	10	172.0–210.0	29	208.0–254.0	8	253.0–288.0	10
F4	36.8–52.7	10	56.0–68.5	29	68.7–89.6	8	66.8–99.5	12
F5	107–122	9	147.0–180.0	28	181.0–220.0	7	212.0–276.0	8
F6	31.3–42.8	9	45.9–57.3	29	51.4–67.6	6	66.3–79.2	8
F7	32.5–44.3	9	46.6–56.5	29	56.8–69.9	6	67.1–79.9	9
F8	63.8–89.4	8	90.6–140.8	20	122.7–135.5	2	139.2–181.0	6
F9	90.7–100.0	8	87.3–142.2	26	150–167	4	182.0–207.0	8
F10	50.9–57.6	5	79.4–98.3	18	96.86–97.4	2	79.9–126.0	5
F11	56.6–70.6	8	82.7–105.3	23	107.9–118.0	5	118.9–149.0	9
F12	221–233	5	233.0–309.0	17	312.0–329.0	3	374.0–426.0	8
F13	228–265	4	250.0–327.0	16	328.0–358.0	3	392.0–445.0	8
F14	231–253	7	232.0–326.0	25	332.0–364.0	3	399.0–453.0	8
F15	196–208	7	244.0–282.0	23	290–350	4	325.0–375.0	9
F16	87.2–109.2	10	121.4–151.6	24	114.0–151.0	3	177.0–202.0	8
F17	74.1–103.9	8	89.7–147.6	24	130.4–132.6	2	156.0–171.0	6
F18	86.5–110.7	8	102.8–148.6	26	143.6–166.0	3	143.0–210.0	7
F19	18.9–27.8	6	20.5–34.8	26	26.3–35.9	6	32.0–44.0	9
F20	8.4–11.7	9	12.7–29.2	26	13.7–23.5	9	18.5–29.6	11

**Table 5.** Tibiotarsal measurements (in mm) for elephant bird species recognized in this study.

measurement	*Mullerornis modestus*, NHMUK A676	*Aepyornis hildebrandti*, MfN MB.AV.70	*Aepyornis maximus*, USNM A605209	*Vorombe titan*, NHMUK A437
Tt1	435.0	473.0	614.0	—
Tt2	20.5	39.3	60.5	75.8
Tt3	85.0	110.0	165.0	206
Tt4	28.2	26.8	39.8	44.3
Tt5	61.0	90.1	129.0	162.0
Tt6	45.5	59.3	84.6	112.5
Tt7	51.0	72.7	100.4	134.5
Tt8	91.0	128.8	184.0	—
Tt9	57.0	85.5	96.5	—
Tt10	65.0	93.9	196.0	—
Tt11	48.0	30.4	55.6	—
Tt12	40.8	65.6	111.9	—
Tt13	57.2	59.2	90.2	—
Tt14	59.8	70.9	105.4	—
Tt16	34.0	53.5	78.9	—
Tt17	28.0	28.0	45.2	—
Tt18	15.2	18.6	30.9	—
Tt19	25.0	43.3	49.7	—
Tt20	63.0	71.6	105.0	—
Tt21	96.0	153.0	225.0	263.0

**Table 6.** Tarsometatarsal measurement ranges for elephant bird species recognized in this study.

measurement	*Mullerornis modestus*		*Aepyornis hildebrandti*		*Aepyornis maximus*		*Vorombe titan*	
	range (mm)	*N*	range (mm)	*N*	range (mm)	*N*	range (mm)	*N*
Tmt1	271.0–324.0	11	288–346	18	352–385	5	419.0–486.0	5
Tmt2	15.0–20.3	11	23.1–29.42	18	27.0–34.7	5	32.0–39.0	5
Tmt3	27.0–32.3	11	50.5–65.0	18	63.3–69.2	5	76.9–87.2	5
Tmt4	27.2–37.6	11	9.2–50.9	17	48.3–54.5	5	59.8–62.7	5
Tmt5	65.0–79.3	11	105.7–118.5	18	125.2–140.2	5	164.0–178.0	5
Tmt6	27.9–39.5	11	34.1–59.9	16	46.4458.6	5	61.6–67.8	5
Tmt7	24.0–30.4	11	31.6–69.3	18	46.4–54.7	5	52.9–68.0	4
Tmt8	26.3–44.7	10	24.5–69.7	18	71.8–87.8	5	74.7–95.3	4
Tmt9	65.8–81.5	11	47.8–123.1	18	140.3–150.5	4	173.0–184.0	5
Tmt10	54.0–67.3	11	78.1–110.6	18	108.3–118.7	4	131.1–153.0	5
Tmt11	64.0–75.3	11	99.8–114.1	18	120.5–140.1	5	161.0–173.0	5
Tmt12	17.6–25.9	11	21.9–31.1	17	32.5–36.8	5	33.9–48.9	5
Tmt13	26.4–34.2	11	35.6–48.5	17	44.6–54.2	5	54.8–61.7	5
Tmt14	21.3–27.4	10	38.4–45.8	18	44.8–55.5	5	59.1–81.6	4
Tmt15	19.7–27.6	11	34.2–39.3	18	43.7–50.7	4	54.9–73.7	4
Tmt16	21.3–27.9	10	38.7–47.3	18	49.8–53.9	4	57.3–66.7	4
Tmt17	30.0–37.2	11	44.26–54.24	16	61–66.1	5	64.1–83.1	4
Tmt18	28.0–34.2	11	38.2–47.1	16	50.7–62.1	5	68.8–76.8	4
Tmt19	26.2–38.2	11	45.6–53.7	16	62.2–68.02	5	76.4–88.1	4
Tmt20	25.4–31.0	11	38.9–52.6	18	50.3–56.2	5	63.7–80.4	4
Tmt21	22.0–25.8	10	32.5–45.8	18	45.4–50.6	5	55.4–89.8	4
Tmt22	19.0–29.9	10	35.7–42.7	18	48–53.5	5	55.2–70.0	4
Tmt23	20.0–34.6	9	47.556.3	18	55.6–70.6	5	61.5–81.4	5
Tmt24	23.0–36.3	10	43.9–53.6	18	52.9–61.7	5	65.2–73.5	5
Tmt25	24.6–33.4	11	35–6.2	18	47.5–58.1	5	59.2–65.3	5
Tmt26	35.0–47.5	9	42.3–61.5	18	55.8–65.1	5	68.6–85.5	5
Tmt27	34.0–68.8	11	50.6–62.1	18	64.6–71.3	5	80.1–96.2	5
Tmt28	24.0–45.6	10	49.5–64.3	17	53.6–73.4	5	77.2–100.1	5
Tmt29	20.5–30.3	10	36.0–55.4	17	43.4–66.1	5	54.2–59.1	5
Tmt30	26.1–33.2	11	40–51.8	18	51.1–56.4	5	53.6–70.6	5
Tmt31	26.8–35.3	11	39.0–48.1	18	49.2–58.9	5	62.4–67.6	5
Tmt32	6.8–12.8	10	8.8–17.2	17	14.5–20.2	5	14.1–24.6	5
Tmt33	56.0–67.4	10	85.5–104.5	18	121.6–135.3	4	151.9–167.0	4
Tmt34	10.2–19.9	8	14.8–25.2	16	21.1–33.4	5	26.1–36.4	3
Tmt35	32.6–45.6	11	56.3–64.6	17	73.2–79.6	4	88.2–99.7	5
Tmt36	19.0–34.2	11	38.5–50.5	17	45.1–53.1	5	50.8–69.8	5
Tmt37	18.2–33.6	8	36.7–53.2	17	37.9–49	4	52.3–82.0	5
Tmt38	38.5–49.0	9	55.7–71.4	17	72.5–76.9	5	73.4–93.0	3
Tmt39	44.0–53.8	9	62.44–76.2	17	84.1–91.7	5	76.5–110.7	4
Tmt40	53.8–65.2	9	82.8–101.1	17	112–120.2	4	127.0–137.0	4
Tmt41	271.0–300.0	11	259–310	17	326–357	5	391.0–440.0	4
Tmt42	261.0–313.0	10	258–333	17	356–372	4	380.0–447.0	5
Tmt43	263.0–317.0	10	274–328	17	363–372	4	415.0–457.0	5
Tmt44	265.0–312.0	10	284–334	17	312–381	5	414.0–459.0	4

### 5.3. Spatio-temporal distribution of Aepyornithidae

**Figure 5.**
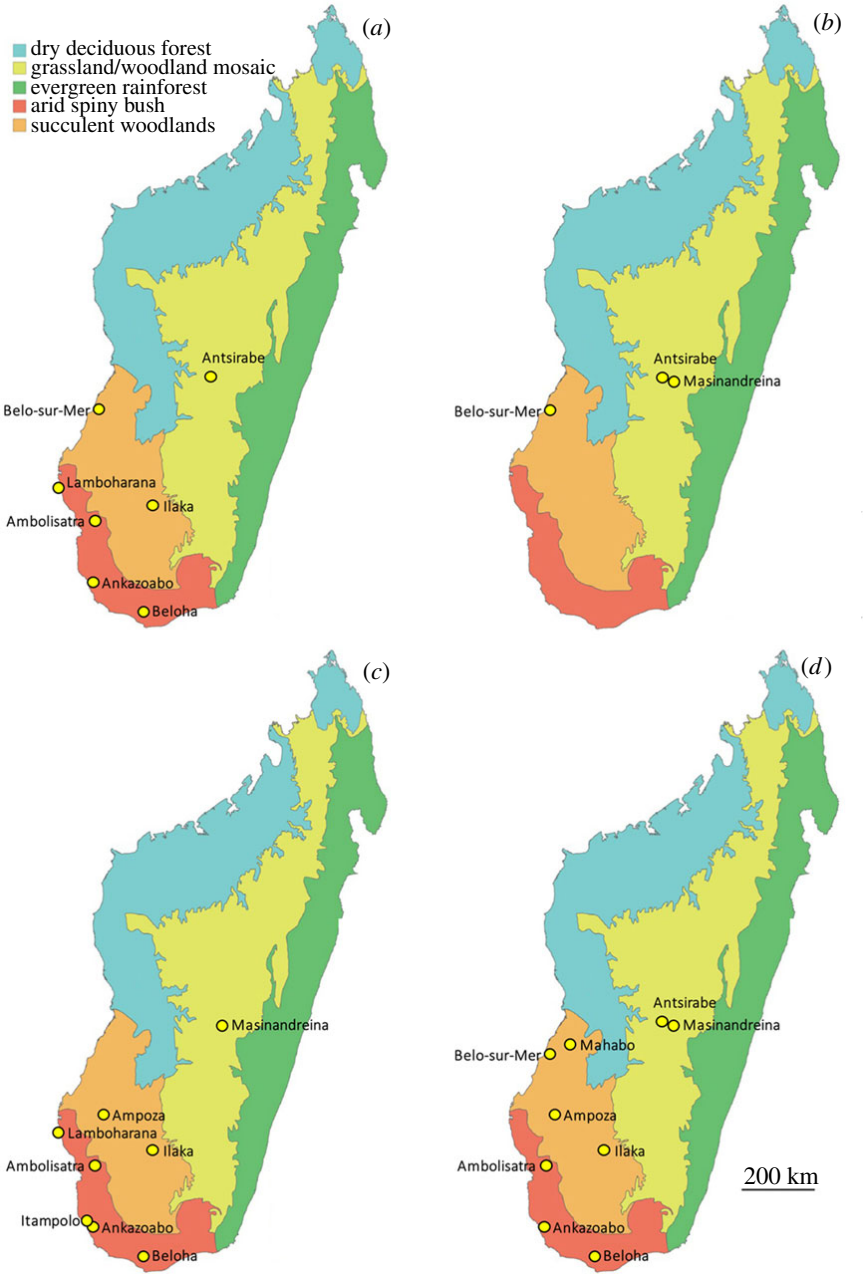
Distribution across Madagascar of identified specimens of elephant bird species recognized in this study. (*a*) *Mullerornis modestus*; (*b*) *Aepyornis hildebrandti*; (*c*) *Aepyornis maximus* and (*d*) *Vorombe titan*.

Owing to the poorly resolved clustering of tibiotarsal data, we selected only femoral and tarsometatarsal geographical location data to reconstruct distributions of newly defined elephant bird taxa. Specimens with high uncertainty were also removed from the pooled location dataset. Locality data associated with well-resolved specimens in our analysis (electronic supplementary information, tables S1 and S2) are plotted by species in figure 5. Our data demonstrate that *Mullerornis modestus*, *Aepyornis maximus* and *Vorombe titan* were widely distributed across Madagascar, and occurred sympatrically across three major ecogeographical zones: arid spiny bush in the south, succulent woodlands in the southwest and grassland/woodland mosaic in the central highlands [53]. Almost all specimens of *Aepyornis hildebrandti* are restricted to the central highlands near Antsirabe and Masinandreina, except for one tarsometatarsus found at Belo-sur-Mer (MNHN MAD 388). This specimen is the type specimen for *Aepyornis lentus* and is missing more than 25% of measurement data, leading to potential unreliability of cluster assignment. New AMS dates for specimens assigned to *Aepyornis hildebrandti* and *Vorombe titan* are given in table 7.

**Table 7.** New direct AMS dates for elephant bird bones assigned to different morphometric clusters.

specimen number	skeletal element	collection locality	species ID	laboratory number	^14^C age (years BP)	calibrated date (95% confidence limits), ±2*σ*
ZIUU 34(A46)	tarsometatarsus	Masinandreina	*Aepyornis hildebrandti*	OxA-34758	1537 ± 25	1420–1314 BP
MNHN MAD 364	femur	Ankazoabo	*Vorombe titan*	OxA-33531	2470 ± 24	2699–2352 BP
NHMUK A2142	femur	Amposa	*Vorombe titan*	OxA-34776	3381 ± 24	3680–3478 BP

## 6. Systematic Palaeontology

**Order Struthioniformes Latham, 1790** [54]

**Family Aepyornithidae Bonaparte, 1853** [11]

**Revised diagnosis:**

**Femur:** Trochanter femoris extremely large, expanded medio-laterally and medio-distally. Crista trochanteris rounded and convex, expanded cranio-caudally and medially, oriented slightly caudally; muscle scars present on lateral facies. Facies articularis antitrochanterica concave. Shaft flattened caudally. Linea intermuscularis caudalis leads to medial margin of shaft. Linea intermuscularis cranialis leads to distal margin of shaft. Impression of m. gastrocnemialis lateralis presents as large, deep pit on cranio-lateral margin, just proximal to very wide trochlea fibularis. Sulcus patellaris broad, deep u-shape in distal aspect, laterally directed.

**Tibiotarsus:** Distally flattened, much wider medio-laterally than cranio-caudally deep. Pronounced crista cnemialis lateralis, expanded proximally. Pronounced crista cnemialis cranialis, expanded medially. Both cnemialis crista oriented medially. Incisura tibialis wide, deep u-shape in proximal aspect, separating facies articularis lateralis and crista cnemialis lateralis. Facies articularis prominent and rounded, longer proximo-distally than latero-medially. Shaft narrows from proximal end, with linea intermuscularis cranialis terminating on lateral margin. Canalis extensoris proximal to condylus lateralis at distal end. Condylus lateralis with greater caudo-cranial expansion than condylus medialis. Pons supratendineus present. Distal end much wider medio-laterally than cranio-caudally.

**Tarsometatarsus:** Proportionately long, with triangular facies dorsalis. Proximal end and trochleae expanded medio-laterally; lateral margin expanded proximally, medial margin enlarged distally. Shaft flattened dorsally. Single high and long hypotarsal ridge. In anterior view, foramina closely spaced within sulcus extensorius formed by flattened-triangular orientation of metatarsi. In posterior view, foramina widely separated by hypotarsal ridge oriented away from midline of broad, long shaft. Distal end with large intertrochlear notches. Three trochleae; trochlea III always terminating furthest distally and marginally forward of shaft and larger than trochleae II and IV, which are nearly equal in size.

**Genus *Aepyornis* Geoffroy Saint-Hilaire, 1851** [10]

*Aepiornis* Geoffroy Saint-Hilaire, 1851, p. 52 [55]

*Epiornis* Muller and Baldamus, 1851, p. 48 [56]

*Epyornis* Bonaparte, 1853, p. 139 [11]

**Type species:** *Aepyornis maximus* Geoffroy Saint-Hilaire, 1851 (by monotypy) [10].

**Recognized species:** *Aepyornis maximus* Geoffroy Saint-Hilaire, 1851 [10]; *Aepyornis hildebrandti* Burckhardt, 1893 [23].

**Revised diagnosis:**

**Femur:** Proportionately broader and more robust than *Mullerornis*, and slightly more robust than *Vorombe*. Facies articularis antitrochanterica is shallow concave surface between trochanter femoris and caput femoris, which are oriented at shallower angles proximally than distally. Significantly larger than *Mullerornis* in following measurements: F1–F4, F6, F10–F12, F16 (after Bonferroni correction of *p*-values, *α* = 0.0026). Significantly smaller than *Vorombe* in following measurements: F1–F14, F16–F17, F19–F20 (after Bonferroni correction of *p*-values, *α* = 0.0026).

**Tibiotarsus:** Shaft broader in proportion to overall size in comparison to other genera. Smaller tibiotarsi (*A. hildebrandti*) of similar length to *Mullerornis* but considerably more robust, with more rounded cnemial crista. Proximal end expanded, particularly medio-laterally. Margin between crista cnemialis cranialis and crista cnemialis lateralis flatter than other genera.

**Tarsometatarsus:** Smaller tarsometatarsi (*A. hildebrandti*) of similar length to *Mullerornis* but medio-laterally broader and with much shallower triangular cross-section. Trochlea IV distally larger and longer than trochlea II. Significantly larger than *Mullerornis* in following measurements: Tmt2–Tmt6, Tmt9–Tmt11, Tmt13–Tmt25, Tmt27–Tmt31, Tmt33–Tmt39 (after Bonferroni correction of *p*-values, *α* = 0.001). Significantly smaller than *Vorombe* in following measurements: Tmt1, Tmt3–Tmt6, Tmt10–Tmt11, Tmt13–Tmt22, Tmt27–Tmt28, Tmt31, Tmt33, Tmt35–Tmt36, Tmt38–Tmt41, Tmt43–Tmt44 (after Bonferroni correction of *p*-values, *α* = 0.001).

**Revised description:**

**Femur:** (In addition to diagnostic features above) Crista trochanteris large, rounded and convex. Medio-distal margin of caput femoris with broad curvature, transitioning into medial margin of shaft. Shaft narrows from proximal end, with straight middle section, and expanding distally into condylus medialis. Condylus medialis expanded proximo-distally. Trochlea fibularis with acute angle to lateral margin of shaft and trochanter femoris. Fossa poplitea with pronounced, proximally arched margin; very large, positioned above lateral portion of condylus medialis and sulcus patellaris.

**Tibiotarsus:** (In addition to diagnostic features above) Crista cnemialis cranialis directed proximo-medially, extending proximally to crista cnemialis lateralis. Rounded, proximally expanded crista cnemialis lateralis extends into ridge, leading into prominent, straight linea intermuscularis cranialis that terminates approximately 50% along length of shaft on lateral margin. Proximal margin of sulcus intercnemialis is very shallow concave curve between the two crista in cranial view. Shaft medially straight and laterally curved, expanding into distal condyles. Distal articular surface broad and shallow.

**Tarsometatarsus:** (In addition to diagnostic features above) Very robust (mean minimum shaft width 8.2% of total length). Medio-laterally broad at proximal end, with rounded lateral portion; expanded plantar-dorsally, transitioning into shaft. Tuberositas m. tibialis cranialis small, rounded, slightly larger medio-laterally than proximo-distally. Shaft very broad, narrowing slightly in medial section, with both medial and lateral margins having continuous broad concave curvatures.

***Aepyornis maximus*** Geoffroy Saint-Hilaire, 1851 [10]

*Aepyornis maximus* Geoffroy Saint-Hilaire, 1851, p. 104 [10]

*Aepyornis medius* Milne-Edwards and Grandidier, 1869, p. 97 [21]

*Aepyornis cursor* Milne-Edwards and Grandidier, 1894, p. 124 [18]

**Syntype series:** Tarsometatarsus of adult individual and two eggs, from ‘the south coast’ of Madagascar, purchased from Merchant Captain M. Abadie. Original tarsometatarsus now cannot be located, and eggs cannot be distinguished from other collections in MNHN.

**Lectotype:** Tarsometatarsus from original syntype series (no allocated specimen number), designated herein.

**Revised diagnosis:**

**Femur:** Compared to *A. hildebrandti*, has similar length and width of proximal and distal ends, but markedly more robust; trochanter femoris larger in proportion to total femur size and more expanded proximally and dorsoventrally; caput femoris slightly shorter medially; shaft with greater circumference and more clearly defined linea intermuscularis cranialis. Significantly larger than *A. hildebrandti* in F15 (after Bonferroni correction of *p*-values, *α* = 0.0026).

**Figure 6.**
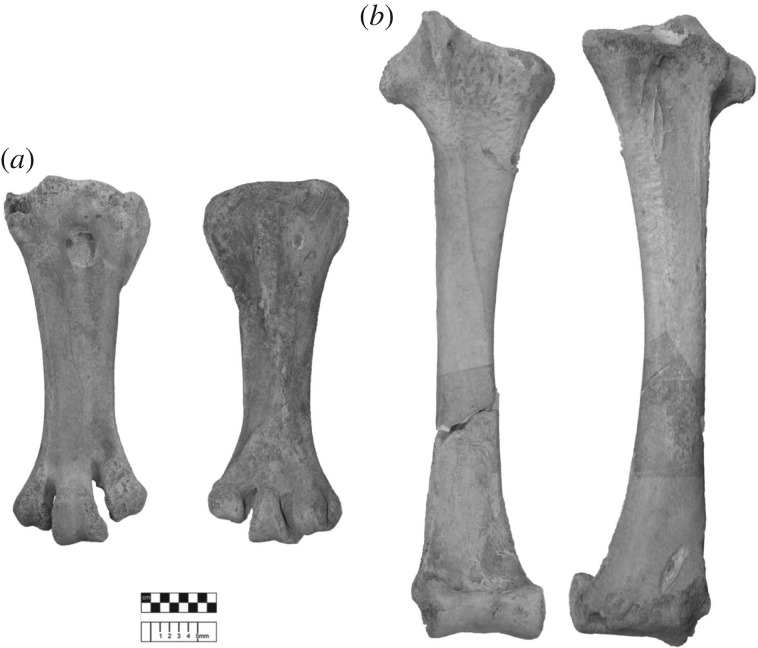
Diagnostic material of *Aepyornis maximus*. (*a*) Tarsometatarsus (USNM A65208), Ilaka, Ambositra, Madagascar. (*b*) Tibiotarsus (USNM A65209), Ilaka, Ambositra, Madagascar.

**Tibiotarsus:** Compared to *A. hildebrandti*, crista cnemialis lateralis more expanded proximally and more laterally oriented, and with shallower angle of transition into pronounced linea intermuscularis cranialis; fascia gastrocnemialis proportionally larger; shaft proportionally wider, and similarly expanded at proximal and distal ends; distal condyles more expanded medio-laterally and protrude equally distally. See figure 6.

**Tarsometatarsus:** Compared to *A. hildebrandti*, proximal end flatter, with less proximal expansion on lateral metatarsal and more expanded medio-laterally; expansion of medial margin continues more distally; trochleae proportionately less expanded*.* Significantly larger than *A. hildebrandti* in following measurements: Tmt9, Tmt14, Tmt19, Tmt33, Tmt35–Tmt36, Tmt40, Tmt43 (after Bonferroni correction of *p*-values, *α* = 0.0012). See figure 6.

**Revised description:**

**Femur:** (In addition to descriptions and diagnostic features above) Comparatively long and very stout (minimum midshaft width 17.3% of total length). Condylus medialis expanded medially and with flatter distal surface leading into trochlea fibularis; fossa poplitea with pronounced, proximally arched margin with slight orientation towards medial shaft margin.

**Tibiotarsus:** (In addition to descriptions and diagnostic features above) Long and very robust (minimum midshaft width 10% of total length). Shaft with distinct curvature on lateral margin.

**Tarsometatarsus:** (In addition to descriptions and diagnostic features above) Long and stout (minimum shaft width 8.2% of total length). Foramina within shallow fossa infracotylaris dorsalis, which has slight concave curvature from proximal margin of articular surface. Proximal end more medio-laterally enlarged and with slightly greater lateral length than distal end.

**Proportions of limb elements:** 1 : 1.6 : 0.9 (tarsometatarsus : tibiotarsus : femur). Data in tables 4–6.

**Measurements of type material (mm) reported by Owen** [52]**:** Extreme breadth across trochlear condyles, 127 mm (5 inches); transverse diameter of shaft 6 inches above lower end, 74 mm (2.9 inches); antero-posterior diameter of shaft 6 inches above lower end, 33 mm (1.3 inches).

***Aepyornis hildebrandti*** Burckhardt, 1893 [23]

*Aepyornis hildebrandti* Burckhardt, 1893, p. 127 [23]

*Aepyornis lentus* Milne-Edwards and Grandidier, 1894, p. 124 [18]

*Aepyornis gracilis* Monnier, 1913, p. 15 [24]

**Syntype series:** Femur (MfN MB.AV.73), tibiotarsus (MfN MB.AV.70), tarsometatarsus (MfN MB.AV.67), from Antsirabe, Madagascar.

**Lectotype:** Tarsometatarsus (MfN MB.AV.67), designated by Brodkorb [28].

**Revised diagnosis:**

**Femur:** Compared to *A. maximus*, trochanter femoris proportionally smaller and less expanded proximally and dorsoventrally; caput femoris longer medially, with more continuous curvature of distal margin; shaft much more slender, with less well-defined linea intermuscularis cranialis. Significantly smaller than *A. maximus* in F15 (after Bonferroni correction of *p*-values, *α* = 0.0026).

**Tibiotarsus:** Compared to *A. maximus*, crista cnemialis lateralis less expanded proximally and more medially oriented, and with more acute angle of transition into weak linea intermuscularis cranialis; concavity from crista cnemialis cranialis to shaft less acute; fascia gastrocnemialis smaller; shaft proportionally narrower, and more expanded at proximal end than at distal end; distal condyles more expanded proximo-distally; condylus lateralis protrudes distally to condylus medialis.

**Tarsometatarsus:** Compared to *A. maximus*, metatarsal IV more expanded proximally, creating angled proximal articular surface; medial margin less expanded distally; trochleae more expanded medio-laterally*.* Significantly smaller than *A. maximus* in following measurements: Tmt9, Tmt14, Tmt19, Tmt33, Tmt35–Tmt36, Tmt40, Tmt43 (after Bonferroni correction of *p*-values, *α* = 0.0012).

**Revised description:**

**Femur:** (In addition to descriptions and diagnostic features above) Comparatively short but robust (minimum midshaft width 16.6% of total length). Condylus medialis expanded proximo-distally; concave fascia leading into trochlea fibularis; fossa poplitea with pronounced, proximally arched margin in centre of shaft.

**Tibiotarsus:** (In addition to descriptions and diagnostic features above) Long and robust (minimum midshaft width 8.3% of total length). Shaft with distinct curvature on lateral margin.

**Tarsometatarsus:** (In addition to descriptions and diagnostic features above) Small and stout (minimum shaft width 8.2% of total length). Foramina within fossa infracotylaris dorsalis, which has concave curvature from proximal margin of articular surface. Distal end more medio-laterally enlarged than proximal end; lateral length greater than medial length.

**Proportions of limb elements:** 1 : 1.5 : 1 (tarsometatarsus : tibiotarsus : femur). Data in tables 4–6.

**Measurements of type material (mm). MfN MB.AV.73:** F2 = 43.24; F3 = 158; F4 = 52.7; F16 = 100.4; F20 = 11.2. **MfN MB.AV.70:** Tt1 = 473; Tt2 = 39.32; Tt3 = 110; Tt4 = 26.76; Tt5 = 90.1; Tt6 = 59.34; Tt7 = 72.7; Tt8 = 128.8; Tt9 = 85.54; Tt10 = 93.92; Tt11 = 30.4; Tt12 = 65.6; Tt13 = 59.16; Tt14 = 70.88; Tt15 = 53.48; Tt16 = 28.02; Tt17 = 18.62; Tt18 = 43.32; Tt19 = 71.6; Tt20 = 153. **MfN MB.AV.67:** Tmt1 = 266; Tmt2 = 21.04; Tmt3 = 42.98; Tmt4 = 41.42; Tmt5 = 96.02; Tmt8 = 51.88; Tmt9 = 97.12; Tmt10 = 68.86; Tmt11 = 96.86; Tmt13 = 40.7; Tmt14 = 32.76; Tmt15 = 31.82; Tmt16 = 36.02; Tmt17 = 44.94; Tmt18 = 40.48; Tmt19 = 45.6; Tmt20 = 36.18; Tmt21 = 31.02; Tmt22 = 33.8; Tmt23 = 42.34; Tmt24 = 40.24; Tmt25 = 37.44; Tmt26 = 42.84; Tmt27 = 45.7; Tmt28 = 45.14; Tmt29 = 34.16; Tmt30 = 41.5; Tmt31 = 36.8; Tmt32 = 13.28; Tmt33 = 80.28; Tmt34 = 19.16; Tmt35 = 52.8; Tmt36 = 38.38; Tmt41 = 252; Tmt42 = 237; Tmt43 = 261; Tmt44 = 256.

**Genus *Mullerornis* Milne-Edwards and Grandidier, 1894** [18]

*Flacourtia* Andrews, 1895, p 23 [20]

**Type species:** *Mullerornis betsilei* Milne-Edwards and Grandidier, 1894 [18]; designated by Richmond [19].

**Recognized species:** *Mullerornis modestus* (Milne-Edwards and Grandidier, 1869) [21].

**Revised diagnosis:**

**Femur:** Smaller, proportionately narrower and less robust than *Aepyornis* or *Vorombe*. Facies articularis antitrochanterica and caput femoris form smooth concave surface, oriented proximo-distally at shallower angle proximally than distally. Distal end medio-laterally expanded. Significantly smaller than *Aepyornis* in following measurements: F1–F4, F6, F10–F12, F16 (after Bonferroni correction of *p*-values, *α* = 0.0026). Significantly smaller than *Vorombe* in following measurements: F1–F14, F16–F17, F19–F20 (after Bonferroni correction of *p*-values, *α* = 0.0026).

**Tibiotarsus:** Similar in total length to *Aepyornis hildebrandti*, but with more slender shaft and well-defined, protruding cnemial crista. Proximal end expanded laterally, but with reduced medial expansion compared to other genera. Crista cnemialis lateralis prominent, projecting proximally, forming distinct curved and laterally positioned ridge. Crista cnemialis cranialis more prominent than in other genera, expanded markedly medially to form extremely pronounced curve into shaft. Margin between crista cnemialis cranialis and crista cnemialis lateralis sharply concave.

**Tarsometatarsus:** Similar in length to *Aepyornis hildebrandti*, but markedly narrower. Shaft with acute triangular cross-section. Trochleae with reduced lateral expansion and minimal medial expansion. Trochlea IV protrudes distal to trochlea II; trochlea III protrudes distal to trochleae II and IV. Significantly smaller than *Aepyornis* in following measurements: Tmt2–Tmt6, Tmt9–Tmt11, Tmt13–Tmt25, Tmt27–Tmt31, Tmt33–Tmt39 (after Bonferroni correction of *p*-values, *α* = 0.001). Significantly smaller than *Vorombe* in following measurements: Tmt1–Tmt6, Tmt9–Tmt36, Tmt38–Tmt41, Tmt43–Tmt44 (after Bonferroni correction of *p*-values, *α* = 0.001).

**Revised description:**

**Femur:** (In addition to descriptions and diagnostic features above) Short and slender (minimum midshaft width 12.7% of total length). Crista trochanterica large, rounded and convex at proximal end. Distal margin of caput femoris with reduced concave curvature. Shaft narrows in middle, curved medially and laterally, expanding into broad condylus medialis; with reduced concave curvature on distal fascia. Medio-distal condyle much less expanded than latero-distal condyle, and protrudes proximally. Fossa poplitea very large in proportion to size of femur, with poorly defined proximal margin, positioned above sulcus patellaris. Trochlea fibularis very large in proportion to size of femur; oriented disto-laterally, pointing away from trochanter femoris.

**Tibiotarsus:** (In addition to descriptions and diagnostic features above) Long and slender (minimum midshaft width 4.7% of total length). Crista cnemialis cranialis extends markedly past crista cnemialis lateralis, directed proximo-medially. Crista cnemialis lateralis protrudes markedly medially, transitioning sharply into clear linea intermuscularis that approaches lateral margin approximately 50% along shaft length, then runs parallel and becomes undefined above condylus lateralis. Proximal margin of sulcus intercnemialis is sharply concave curve between the two crista in cranial view. Shaft relatively straight, narrowing markedly on medial margin but with only shallow curvature on lateral margin and only minor expansion into distal condyles. Distal condyles protrude equally at distal end.

**Tarsometatarsus:** (In addition to descriptions and diagnostic features above) Small and slender (minimum shaft width 5.7% of total length). Proximal end with small amount of lateral expansion and marginal medial expansion. Hypotarsal ridge very broad and deep in proximal aspect. Proximal fascia relatively flat, with minimal proximo-distal expansion. Foramina within fossa infracotylaris dorsalis that has concave curvature from proximal margin of articular surface. Tuberositas m. tibialis cranialis centrally positioned, rounded and slightly larger medio-laterally than proximo-distally. Shaft narrow with lateral margin reducing towards distal end, and relatively straight medial margin.

***Mullerornis modestus*** (Milne-Edwards and Grandidier, 1869) [21]

*Aepyornis modestus* Milne-Edwards and Grandidier, 1869, p. 314 [21]

*Mullerornis agilis* Milne-Edwards and Grandidier, 1894, p. 125 [18]

*Mullerornis betsilei* Milne-Edwards and Grandidier, 1894, p. 125 [18]

*Mullerornis rudis* Milne-Edwards and Grandidier, 1894, p. 125 [18]

**Holotype:** Femur (MNHN 1908-5), from Ambolisatra, Madagascar.

**Revised diagnosis:** As for genus.

**Revised description:** As for genus.

**Proportions of limb elements:** 1 : 1.5 : 0.9 (tarsometatarsus : tibiotarsus : femur). Data are presented in tables 4–6.

**Measurements of type material (mm). MNHN 1908-5:** F1 = 255; F2 = 29.9; F3 = 121; F4 = 41.84; F5 = 112; F6 = 34.9; F7 = 34.54; F8 = 68.54; F9 = 90.66; F10 = 55.98; F11 = 63.66; F12 = 223; F14 = 240; F16 = 96.52; F17 = 75.88; F18 = 95.24; F19 = 23.36; F20 = 11.66.

**Genus *Vorombe* gen. nov.**

**Etymology:** From the Malagasy for ‘big bird’ (neuter).

**Type species:** *Aepyornis titan* Andrews, 1894 [22]

**Recognized species:** *Vorombe titan* (Andrews, 1894) [22]

**Diagnosis:**

**Femur:** Extremely large and robust in comparison to other genera, with enlarged proximal and distal ends. Medio-distal margin of caput femoris with more acute curvature than in other genera. Facies antitrochanterica and caput femoris form smooth concave surface. Caput femoris oriented at equal angles perpendicular to shaft proximo-distally. Marked crista supracondylaris medialis present (absent in other genera). Condylus medialis expanded medially and flatter than in *Aepyornis*. Significantly larger than both *Aepyornis* and *Mullerornis* in all measurements (after Bonferroni correction of *p*-values, *α* = 0.0026).

**Tibiotarsus:** Extremely large in comparison to other genera. Proximal and distal ends enlarged, particularly medio-laterally, with proximal articular surface marginally more concave than *Aepyornis* but much less than *Mullerornis*, and with more pronounced narrowing transition into shaft; shaft narrower in proportion to total length compared to *Aepyornis.* Lateral condyle markedly more expanded distally and laterally than in other genera, terminating distal to condylus medialis.

**Tarsometatarsus:** Considerably larger and markedly more expanded medio-laterally than other genera, particularly at proximal and distal ends. Lateral portion of proximal articular surface protrudes proximally to medial portion, creating markedly angled proximal articular surface similar to *A. hildebrandti.* Trochlea II protrudes marginally proximal to trochlea IV. Trochleae II and IV more equal in size than in other genera; expanded similarly both medio-laterally and dorsoventrally. Significantly larger than *Mullerornis* in all measurements (after Bonferroni correction of *p*-values, *α* = 0.001). Significantly larger than *Aepyornis* in following measurements: Tmt1, Tmt3–Tmt6, Tmt10–Tmt11, Tmt13–Tmt22, Tmt27–Tmt28, Tmt31, Tmt33, Tmt35–Tmt36, Tmt38–Tmt41, Tmt43–Tmt44 (after Bonferroni correction of *p*-values, *α* = 0.001).

**Description:**

**Femur:** (In addition to descriptions and diagnostic features above) Robust (minimum midshaft width 16.3% of total length). Crista trochanterica large, rounded and convex. Medio-distal margin of caput femoris transitions into medial margin of narrowing, medially straight shaft, which then expands into condylus medialis*.* Condylus lateralis expanded proximally. Trochlea fibularis very large, shallow and broad; parallel to shaft and trochanter femoris. Fossa poplitea with poorly defined proximal margin; transitions smoothly into shaft, positioned above lateral portion of condylus medialis and sulcus patellaris.

**Tibiotarsus:** (In addition to descriptions and diagnostic features above) Very long (minimum midshaft width 7.9% of total length). Crista cnemialis cranialis extends past crista cnemialis lateralis, directed proximo-medially. Crista cnemialis lateralis rounded, medially and marginally proximally expanded; transitions via smooth curve into medial surface of shaft, extending into prominent, straight and well-defined linea intermuscularis terminating on lateral margin just proximal to distal condyles. Proximal margin of sulcus intercnemialis very shallow concave curve between the two crista in cranial view. Shaft narrowing near proximal end on medial margin, but with only shallow curvature on lateral margin, becoming very straight and parallel at midshaft before expanding markedly into distal condyles.

**Tarsometatarsus:** (In addition to descriptions and diagnostic features above) Robust (minimum shaft width 7.9% of total length) and long. Extremely medio-laterally broad at proximal ends; lateral portion rounded and expanded plantar-dorsally. Hypotarsal ridge very broad and deep in proximal aspect. Foramina within shallow fossa infracotylaris dorsalis that has slight concave curvature from proximal margin of articular surface. Tuberositas m. tibialis cranialis small, rounded, slightly larger medio-laterally than proximo-distally. Shaft highly tapered and broad; medial margin becoming straight, lateral margin retains continuous broad concave curvature.

***Vorombe titan*** (Andrews 1894) [22]

*Aepyornis titan* Andrews 1894, p. 18 [22]

*Aepyornis ingens* Milne-Edwards and Grandidier, 1894, p. 124 [18]

**Figure 7.**
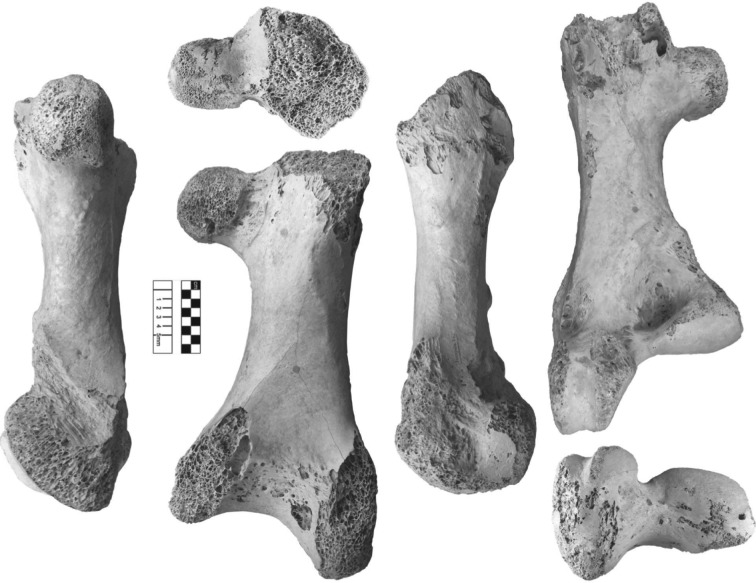
*Vorombe titan*, femur (NHMUK A439), Itampolo (Itampulu Vé), Madagascar; part of syntype series.

**Figure 8.**
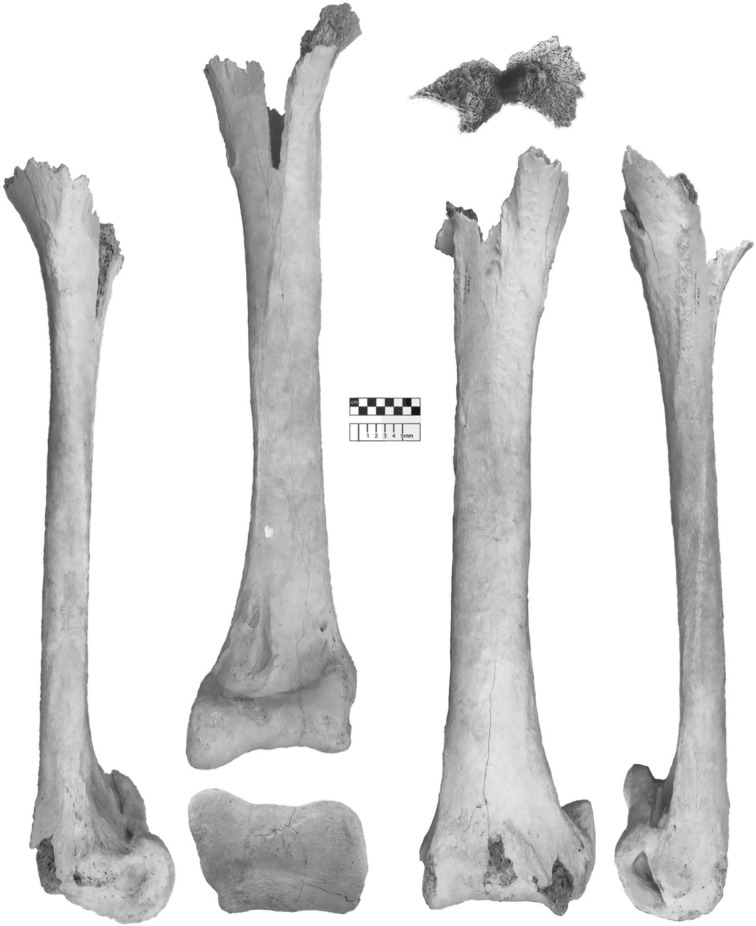
*Vorombe titan*, tibiotarsus (NHMUK A437), Itampolo (Itampulu Vé), Madagascar; part of syntype series.

**Syntype series:** Femur (NHMUK A439), tibiotarsus (NHMUK A437), from Itampolo (Itampulu Vé), Madagascar (figures 7 and 8).

**Lectotype:** Femur (NHMUK A439); newly designated (figure 7).

**Diagnosis:** As for genus.

**Description:** As for genus.

**Proportions of limb elements:** 1 : 1.8 : 1 (tarsometatarsus : tibiotarsus : femur). Data are summarized in tables 4–6.

**Measurements of type material (mm). NHMUK A439:** F2 = 71; F3 = 271; F4 = 91.5; F5 = 232; F6 = 68.6; F7 = 74; F9 = 203; F11 = 141; F14 = 414; F15 = 346; F19 = 32; F20 = 24. **NHMUK A437:** Tt2 = 75.8; Tt3 = 206; Tt4 = 44.3; Tt5 = 162; Tt6 = 112.5; Tt7 = 134.5; Tt20 = 263.

## 7. Discussion

Our study provides the first rigorous quantitative analysis of morphometric variation within elephant birds, using data from almost all of the specimens available for study in global museum collections, and employing multivariate analyses of morphometric data with methods for estimating missing values that are robust to potential sources of error. This exhaustive analysis fundamentally revises the taxonomic framework for understanding diversity and variation within elephant birds, compared to historical taxonomic reviews that were based largely on qualitative assessment of much smaller sample sizes of specimens. We demonstrate that three main morphometric clusters can be identified within measurement data for elephant bird appendicular elements, with one cluster further divisible into two separate subclusters. As one of these clusters (cluster 1, corresponding to *Mullerornis* samples) represents specimens that are already uncontroversially recognized as being distinct at the genus level from the other clusters, the comparably morphometrically distinct largest-bodied cluster (cluster 3) must therefore also be recognized as taxonomically distinct at the genus level. Further morphometric subdivision within cluster 2 is interpreted as representing species-level differentiation. We therefore identify three valid elephant bird genera, two of which are monotypic, and one of which contains two species.

Our new data-driven taxonomic revision recognizes both different numbers and different identities of elephant bird taxa compared to previous assessments. Our taxonomic framework recognizes only four elephant bird species, substantially reducing the number of valid species recognized by earlier authors, who variously identified 15 different putative species (tables 1 and 2); for example, Monnier's taxonomic assessment recognized four species of *Aepyornis* [24], Lamberton recognized five species (two *Aepyornis* spp. and three *Mullerornis* spp.) [25] and Brodkorb recognized seven species (four *Aepyornis* spp. and three *Mullerornis* spp.) [28]. As our revision is based on multivariate analysis of the distribution of variation within and between morphotype clusters in multidimensional shape-space, we consider our taxonomic conclusions to be substantially more robust than previous studies. As all linear measurements used in this study were normalized to the unit variance of the measured features, we were able to control for size biases of major dimensions (e.g. total length) during analysis, allowing the detection of distinct morphometric groups. However, we note that it is possible that our taxonomic hypothesis may represent a conservative estimate of elephant bird species richness based on the limitations of what morphology-based quantitative analysis can resolve, and we encourage further investigation of variation across elephant birds using alternative approaches, such as ancient DNA analysis of well-provenanced material associated with different morphometric clusters, an approach that led to a revision of morphology-based taxonomy in moa [57].

The three genera and four species of elephant birds that we recognize in this study also represent different taxonomic concepts to those recognized by previous authors. The small-bodied genus *Mullerornis* has generally been interpreted in recent decades as comprising three species, *M. agilis*, *M. betsilei* and *M. rudis*. However, not only do we synonymize these three taxa as representing a single species on the basis of morphometric analysis, but we also identify the name *M. modestus* as the senior synonym for all three taxa; this name was previously considered to be a junior synonym of *Aepyornis maximus* [28]. *Aepyornis maximus* has commonly been interpreted as the largest elephant bird, both in older taxonomic reviews and also in popular culture, but the type material of this first elephant bird to be described has rarely been considered since its original description, with the species concept of *A. maximus* instead becoming associated with later collections of very large elephant bird bones that have been erroneously assigned to the taxon. Our analysis demonstrates that the name *Aepyornis* is in fact not associated with the largest known elephant bird material, but instead represents the medium-sized genus-level cluster in our morphometric analysis, with this genus containing only two diagnosable species (*A. hildebrandti* and *A. maximus*) compared with previous assumptions of four or more congeners (table 1).

As the name *Aepyornis* cannot be applied to the largest-bodied genus-level cluster recognized in our analysis, the largest of the elephant birds, for which the names *Aepyornis titan* and *Aepyornis ingens* are available, are here allocated to the new genus *Vorombe*. All body mass estimates for giant extinct birds should be interpreted with caution as they fall outside the range of extant birds used in model construction; however, our newly derived mass estimates for elephant birds based on least femoral shaft circumference measurements (table 3) demonstrate that the mass of *Vorombe* (mean = 642.9 kg, range = 536–732 kg) exceeds estimates based on comparable data for other extinct Quaternary giant birds such as *Dinornis* (Dinornithiformes: range = 61–275 kg) and *Dromornis* (Gastornithiformes: male mean = 583.6 kg, range = 439.3–727.8 kg; female mean = 440.7 kg, range = 316.6–560.0 kg) [58,59], giving it the largest estimated body mass of any bird on record. Indeed, the largest elephant bird femur measured for this study (MNHN MAD 368) was incomplete and therefore could not formally be assigned to a cluster due to our conservative analytical framework, but must also be referable to *Vorombe* on the basis of size; this specimen had a least-shaft circumference of 308 mm and a corresponding mass estimate of 860 kg, making this the largest known bird individual ever recorded. This body mass estimate is comparable to or greater than available estimates for the smallest sauropod dinosaurs (*Europasaurus*: 690 kg; *Magyarosaurus*: 700–1000 kg) [60]. However, prior to our study, the world's largest birds have rarely even been recognized as a distinct species let alone as a separate genus and have instead been generally misinterpreted as merely representing the upper end of variation within *Aepyornis maximus* based on broad, qualitative size ranges assumed for this ‘wastebasket taxon’, leading to underestimation of the true size of the largest elephant birds by previous authors [59].

Allochronic body size reduction across the Pleistocene–Holocene transition, representing an anagenetic response to major environmental and vegetational shifts between glacial and interglacial conditions, is documented in many large-bodied vertebrate lineages [61–63] including moa [42] and other birds [64]. The existence of distinct allochronic Quaternary size morphs within a single evolving lineage can confound interpretation of morphometric variation [42,61,64], and so it is necessary to control or account for sample age in taxonomic studies of Quaternary collections. Unfortunately, dating of elephant bird material has been limited, with most available direct dates reported from eggshell rather than from taxonomically diagnostic skeletal elements [34,65], and there is a need for greatly improved dating to understand the temporal contexts of available samples and known sites. However, we are able to demonstrate that all four of the morphometric clusters we recognize in this study include specimens that are Holocene in age. Recently published direct AMS dates are now available for specimens that we have assigned to *Aepyornis maximus* (USNM A65209, 9428 ± 53 and 9535 ± 70 BP; figure 6) and *Mullerornis modestus* (MNHN MAD 6768, 5597 ± 40 BP) [66]. New direct AMS dates reported here for both *Aepyornis hildebrandti* and *Vorombe titan* demonstrate that specimens assigned to these clusters are also Holocene in age (table 7). We can therefore conclude with confidence that morphometric differentiation seen in elephant birds represents cladogenesis across deep time rather than anagenesis across near time.

Morphological variation in the giant moa *Dinornis*, which was formerly interpreted as representing taxonomic variation, has been shown instead to constitute extreme reversed sexual size dimorphism [57,67], and most extant ratites also exhibit varying levels of sexual size dimorphism [68]. Several authors have hypothesized that elephant birds might have also exhibited sexual size dimorphism, and it has even been suggested that *Aepyornis maximus* and *A*. *medius*, two formerly recognized species that were considered to be distinguishable only by size, could represent male and female morphs of the same species [59]. Our quantitative assessment groups these two putative species within one cluster, and therefore we consider that these supposedly distinct forms are better interpreted as representing natural variation (potentially sexual variation) within a single morphotype*.* Indeed, although we do not exclude the possibility that elephant birds exhibited sexual size dimorphism, our morphometric clusters are scaled and therefore independent of size, and are differentiated by more complex patterns of variation across a large series of characters that would not be expected from sexual size dimorphism. Any sexual size dimorphism is therefore likely to be captured as within-cluster variation in our analysis, and our clusters are better interpreted as representing distinct taxonomic units. Recent quantitative analysis has similarly failed to detect any reliable morphometric differentiation of sexual dimorphs in non-avian dinosaurs [69]. However, we encourage further research to test our new morphotype-based taxonomic framework for aepyornithids, especially through the use of ancient biomolecular techniques or systematic investigation of sex-specific medullary bone formation, to assess whether any observed variation can be associated with sexual dimorphism [59,67,70].

Locality data associated with elephant bird specimens included in distinct morphometric clusters demonstrate the sympatric co-occurrence of *M. modestus*, *A. maximus* and *V. titan* in the south and southwest of Madagascar and into the central highlands. The substantial disparity in size between these different taxa suggests that these birds were able to coexist by exploiting distinct dietary niches and floral interactions [33,71]. However, if the incomplete holotype tarsometatarsus of ‘*Aepyornis lentus*’ is excluded from biogeographical consideration due to potential unreliability of cluster assignment, all of the specimens assigned to *A. hildebrandti* in our analysis are restricted to the highest elevations of the central highlands at Antsirabe and Masinandreina. This biogeographical pattern suggests that, whereas different elephant bird genera were morphologically and ecologically distinct enough to be able to coexist in the same landscapes, different species within the same genus (*Aepyornis*) displayed largely allopatric differentiation between different ecoregions. This spatial pattern is also shown in many other vertebrate taxa across Madagascar today [53], and similar elevational niche differentiation between lowland and highland specialists is also seen in many large-bodied mammalian herbivore guilds [72]. Although populations of the giant moa *Dinornis* that exhibited size differences across altitudinal gradients and habitat types have been shown to be conspecific through ancient DNA analysis [58], comparable species-level differentiation between low-altitude and high-altitude populations is also seen in the emeid moa genus *Pachyornis* on New Zealand's South Island, with *P. elephantopus* occurring in lowland habitats and *P. australis* restricted to subalpine shrublands and fellfields during the Holocene [42]. The allopatric spatial distribution pattern between different recognized species of *Aepyornis* therefore provides further support for our interpretation of clusters 2a and 2b as representing taxonomic variation rather than sexual dimorphism.

Previous assumptions of elephant bird species richness (15 putative proposed species variously accepted by different authors; table 1) are similar to species richness in the other late Quaternary insular radiation of now-extinct ratites, the moa of New Zealand, in which nine valid species in six genera are currently recognized from Holocene deposits [73]. Moa taxa were ecologically differentiated by environmental factors including habitat type and elevation [42]. However, the revised levels of elephant bird species richness presented in this study are substantially lower than for moa. This disparity may partly reflect variation in collection effort and number of available specimens between these two island systems. Madagascar's considerably larger area and greater range of biodiverse ecoregions might be expected to have driven greater local endemism and diversification in ratites than in New Zealand [53], but available elephant bird collections are largely restricted to material from southern Madagascar and the central highlands; however, eggshell remains from archaeological and palaeontological deposits in the extreme north of the island, not associated with skeletal material, indicate that elephant birds were more widely distributed in other ecoregions across the island that are known to contain other locally endemic taxa [30]. Conversely, New Zealand's ecosystems experienced specific geological disruptions during the Cenozoic that are likely to have driven increased diversification in moa, including separation of landmasses (associated with allopatric differentiation between North Island and South Island moa taxa), glacial progression and recession, and tectonic activity [74]. Whereas birds were the only large-bodied terrestrial vertebrates in New Zealand before human arrival, Madagascar's Quaternary ecosystems also contained a series of other large-bodied non-avian terrestrial herbivores (giant lemurs, giant tortoises and hippos), which are likely to have limited the range of niches that elephant birds could occupy and therefore probably restricted diversification in the group.

We encourage further investigation of elephant bird systematics and taxonomy, employing complementary data and methods to those presented in this study. In particular, the suggested bimodality in thickness of elephant bird eggshell [30] was consistent with previous recognition of two size-differentiated elephant bird genera, but becomes more difficult to interpret taxonomically following recognition of three distinct genera, and necessitates rigorous quantitative assessment of patterns of eggshell thickness together with more detailed consideration of eggshell pore morphology and other characters, and efforts to link ancient DNA from eggshells and skeletal remains. We also encourage new investigation of variation in elephant bird cranial characters to test whether our taxonomic hypotheses based on postcranial skeletal elements are borne out by other available skeletal data. However, the new taxonomic framework for the Aepyornithidae that we present here provides an important baseline for future studies of avian evolution and Quaternary ecology, and represents a new framework for understanding Madagascar's past ecosystems and reconstructing extinction chronologies for the island's unique and fascinating megafauna.

## Supplementary Material

TABLE S1. AEPYORNITHID LIMB BONE SPECIMENS AND MEASUREMENTS INCLUDED IN MORPHOMETRIC ANALYSIS

## Supplementary Material

TABLE S2. PCA LOADINGS AND EIGENVALUES FOR MORPHOMETRIC ANALYSES

## Supplementary Material

TABLE S3. PCA LOADINGS AND EIGENVALUES FOR LOG-TRANSFORMED MORPHOMETRIC ANALYSES

## Supplementary Material

FIGURE S1. UNSUPERVISED CLUSTERS OF LOG-TRANSFORMED DATA

## Data accessibility

The datasets supporting this article have been uploaded as part of the electronic supplementary material.
