# Correction to ‘Unexpected diversity within the extinct elephant birds (Aves: Aepyornithidae) and a new identity for the world's largest bird’

**DOI:** 10.1098/rsos.201358

**Published:** 2020-09-16

**Authors:** James P. Hansford, Samuel T. Turvey

**Affiliations:** 1 Institute of Zoology, Zoological Society of London, Regent's Park, London NW1 4RY, UK; 2 Ocean and Earth Science, National Oceanography Centre Southampton, University of Southampton, Waterfront Campus, European Way, Southampton SO14 3ZH, UK

*R. Soc. Open. Sci*. **5**, 181295. (Published 26 September 2018). (doi:10.1098/rsos.181295)

## 1. Introduction

This correction is to fulfil the requirements of the International Commission on Zoological Nomenclature (ICZN) article 8.5.3 [1] for the publication of new taxonomic names. In order for the genus *Vorombe* to be an available nomen, this name needed to be registered in ZooBank at the time of publication, with the ZooBank number appearing with the publication [2]. This correction aims to solve this issue, and the ZooBank LSID number is shown below along with a reiteration of the systematic section. The original work [2] should be cited along with this correction when citing this genus name.

## 2. ZooBank LSID

New genus: *Vorombe*

urn:lsid:zoobank.org:act:474DC517-8346-48D5-8280-67F8A37ABBBF

## 3. Systematic Palaeontology

Order Struthioniformes Latham, 1790 [3]

Family Aepyornithidae Bonaparte, 1853 [4]

Genus *Vorombe* gen. nov.

**Etymology:** From the Malagasy for ‘big bird’ (neuter).

**Type species:** *Aepyornis titan* Andrews, 1894 [5]

**Recognized species:** *Vorombe titan* (Andrews, 1894) [5]

**Diagnosis:**

**Femur:** Extremely large and robust in comparison to other genera, with enlarged proximal and distal ends. Medio-distal margin of caput femoris with more acute curvature than in other genera. Facies antitrochanterica and caput femoris form smooth concave surface. Caput femoris oriented at equal angles perpendicular to shaft proximo-distally. Marked crista supracondylaris medialis present (absent in other genera). Condylus medialis expanded medially and flatter than in *Aepyornis*. Significantly larger than both *Aepyornis* and *Mullerornis* in all measurements.

**Tibiotarsus:** Extremely large in comparison to other genera. Proximal and distal ends enlarged, particularly medio-laterally, with proximal articular surface marginally more concave than *Aepyornis* but much less than *Mullerornis*, and with more pronounced narrowing transition into shaft; shaft narrower in proportion to total length compared to *Aepyornis*. Lateral condyle markedly more expanded distally and laterally than in other genera, terminating distal to condylus medialis.

**Tarsometatarsus:** Considerably larger and markedly more expanded medio-laterally than other genera, particularly at proximal and distal ends. Lateral portion of proximal articular surface protrudes proximally to medial portion, creating markedly angled proximal articular surface similar to *Aepyornis hildebrandti*. Trochlea II protrudes marginally proximal to trochlea IV. Trochleae II and IV more equal in size than in other genera; expanded similarly both medio-laterally and dorsoventrally. Significantly larger than *Mullerornis* in all measurements; significantly larger than *Aepyornis* in following measurements: Tmt1, Tmt3–Tmt6, Tmt10– Tmt11, Tmt13–Tmt22, Tmt27–Tmt28, Tmt31, Tmt33, Tmt35–Tmt36, Tmt38–Tmt41, Tmt43–Tmt44 (see Hansford and Turvey [2] for explanation of measurements).

**Description:**

**Femur:** (In addition to descriptions and diagnostic features above) Robust (minimum midshaft width 16.3% of total length). Crista trochanterica large, rounded and convex. Medio-distal margin of caput femoris transitions into medial margin of narrowing, medially straight shaft, which then expands into condylus medialis. Condylus lateralis expanded proximally. Trochlea fibularis very large, shallow and broad; parallel to shaft and trochanter femoris. Fossa poplitea with poorly defined proximal margin; transitions smoothly into shaft, positioned above lateral portion of condylus medialis and sulcus patellaris.

**Tibiotarsus:** (In addition to descriptions and diagnostic features above) Very long (minimum midshaft width 7.9% of total length). Crista cnemialis cranialis extends past crista cnemialis lateralis, directed proximo-medially. Crista cnemialis lateralis rounded, medially and marginally proximally expanded; transitions via smooth curve into medial surface of shaft, extending into prominent, straight and well-defined linea intermuscularis terminating on lateral margin just proximal to distal condyles. Proximal margin of sulcus intercnemialis very shallow concave curve between the two crista in cranial view. Shaft narrowing near proximal end on medial margin, but with only shallow curvature on lateral margin, becoming very straight and parallel at midshaft before expanding markedly into distal condyles.

**Tarsometatarsus:** (In addition to descriptions and diagnostic features above) Robust (minimum shaft width 7.9% of total length) and long. Extremely medio-laterally broad at proximal ends; lateral portion rounded and expanded plantar-dorsally. Hypotarsal ridge very broad and deep in proximal aspect. Foramina within shallow fossa infracotylaris dorsalis that has slight concave curvature from proximal margin of articular surface. Tuberositas m. tibialis cranialis small, rounded, slightly larger medio-laterally than proximo-distally. Shaft highly tapered and broad; medial margin becoming straight, lateral margin retains continuous broad concave curvature.
