# Supplementary material for: Unexpected diversity within the extinct elephant birds (Aves: Aepyornithidae) and a new identity for the world's largest bird
Source: R Soc Open Sci. 2018 Sep 26;5(9):181295. doi: 10.1098/rsos.181295 (PMC6170582; doi:10.1098/rsos.181295)
Supplement: FIGURE S1. UNSUPERVISED CLUSTERS OF LOG-TRANSFORMED DATA [file rsos181295supp4.pdf]

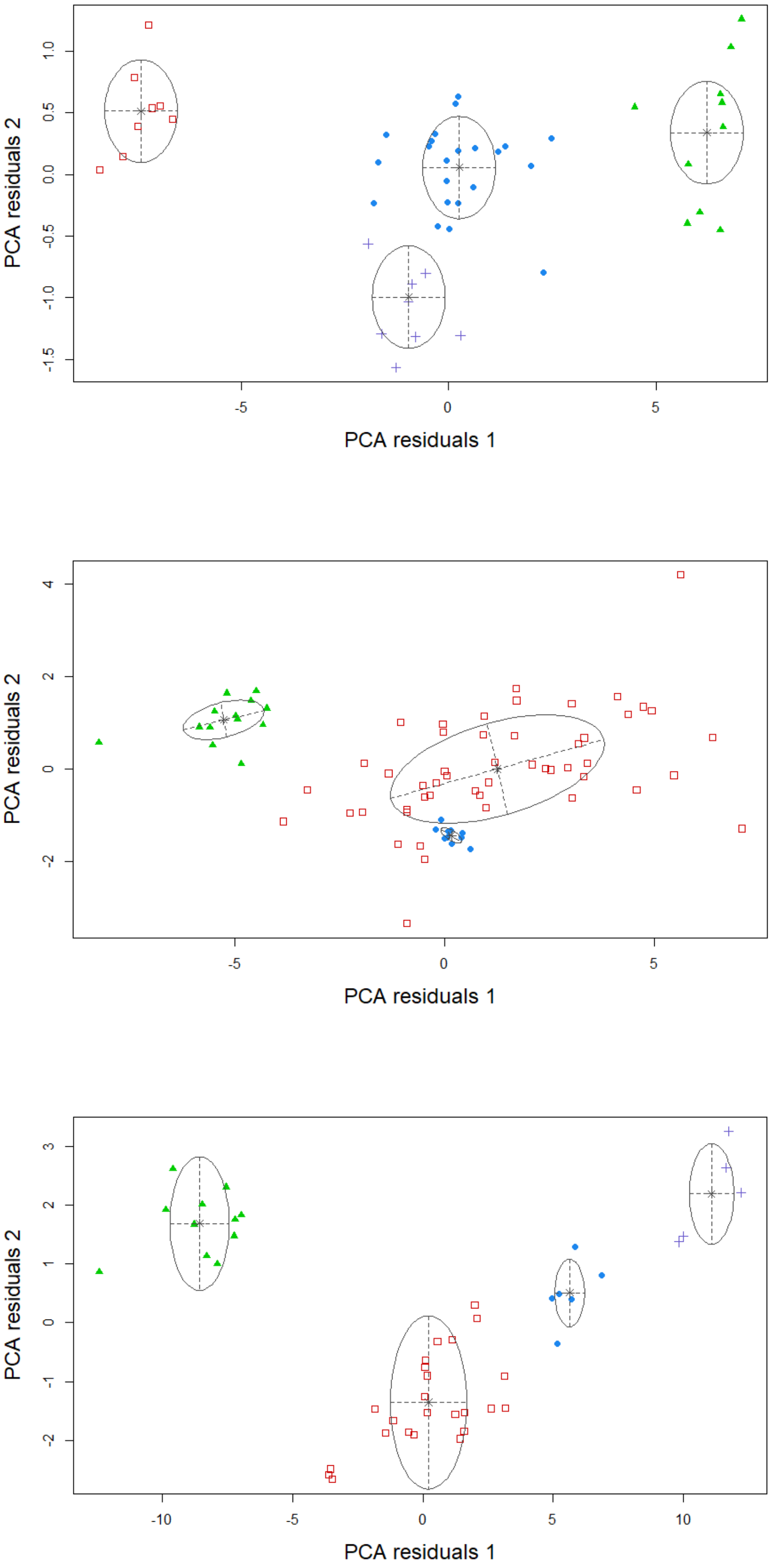

Supplementary Figure S1. Top, Unsupervised PCA clusters of log-transformed femoral data, <25% missing data. Axis 1, 91%; Axis 2, 2.1%. **Middle,** Unsupervised PCA clusters of log-transformed tibiotarsal data, <25% missing data. Axis 1, 56%; Axis 2, 7.6%. **Bottom,** Unsupervised PCA clusters of log-transformed tarsometatarsal data, <25% missing data. Axis 1, 85.9%; Axis 2, 6%.
